# First-time report on compound isolation from two *Colocasia* species: vegetable-derived bioactive metabolites and their medicinal potential

**DOI:** 10.3389/fphar.2024.1474706

**Published:** 2024-12-04

**Authors:** Safaet Alam, Fahmida Tasnim Richi, Nazim Uddin Emon, Abu Asad Chowdhury, Choudhury Mahmood Hasan, Mohammad Rashedul Haque

**Affiliations:** ^1^ Chemical Research Division, BCSIR Dhaka Laboratories, Bangladesh Council of Scientific and Industrial Research (BCSIR), Dhaka, Bangladesh; ^2^ Department of Pharmaceutical Chemistry, Faculty of Pharmacy, University of Dhaka, Dhaka, Bangladesh; ^3^ Department of Pharmacy, Faculty of Science and Engineering, International Islamic University Chittagong, Chittagong, Bangladesh

**Keywords:** *Colocasia gigantea*, *Colocasia affinis*, vegetable, NMR, antibacterial, antidiarrheal, analgesic, anti-inflammatory

## Abstract

**Background:**

*Colocasia affinis* Schott and *Colocasia gigantea* Hook.f. are two commonly found vegetable species of the genus *Colocasia*, found mainly in the Asian region.

**Objectives:**

The objective of this study was to isolate bioactive phytochemicals from *C. affinis* and *C. gigantea* and elucidate their structure employing the NMR technique followed by bioactivity evaluation.

**Methodology:**

Column chromatography was utilized to isolate phytochemicals, followed by NMR analysis for characterization. An *in vivo* analgesic test was performed through an acetic acid-induced writhing test, an anti-inflammatory test was performed through a formalin-induced licking test, and an antidiarrheal test was performed through a castor oil-induced diarrhea model. The *in vitro* antimicrobial test was executed through the disc diffusion method. Computer-aided simulation was also implemented to validate the wet laboratory results.

**Results:**

Six compounds from *C. affinis* and *C. gigantea* were isolated and characterized from the dichloromethane (DCM)-soluble fractions of the methanolic extracts of these two species. Three of the compounds were from *C. gigantea* and proposed as penduletin **(C1)**, a mixture of α-amyrin **(C2a)**, β-amyrin **(C2b)**, and monoglyceride of stearic acid **(C3),** while the remaining three compounds were from *C. affinis* and proposed as penduletin **(C4)** (which was also isolated from *C. gigantea*), 7,8-(3″,3″-dimethyl-pyrano)-4′-hydroxy flavonol **(C5)**, and lastly a mixture of 7,8-(3″,3″-dimethyl-pyrano)-4′-hydroxy flavonol **(C5)** and 4′,7,8-trihydroxy flavonol **(C6)**. These compounds showed promising zones of inhibition against Gram-positive and Gram-negative bacteria and fungi. In the antidiarrheal test, **C5** demonstrated the highest reduction in castor oil-induced diarrhea (44.44%) at a dose of 20 mg/kg compared to loperamide’s 77.78% reduction. However, the analgesic assessment showed a noteworthy inhibition of acetic acid-induced writhing by **C1/C4** and **C2** (56.52%) at a dose of 20 mg/kg compared to the 76.09% by diclofenac sodium. In comparison, **C2** showed pronounced anti-inflammatory action by 68.15% and 52.06% reduction, respectively, in the early and later phases compared to the ibuprofen’s outcomes of 73.54% and 74.68%. Plausible targets such as dihydrofolate reductase (DHFR) for antimicrobial, kappa opioid receptor (KOR) for antidiarrheal, and cyclooxygenase 2 (COX-2) for anti-inflammatory and analgesic activities showed a noteworthy binding affinity with isolated compounds, and ADME/T studies displayed these phytochemicals’ drug-likeness profiles.

**Conclusion:**

To the best of our knowledge, this is the first report on compound isolation from these plants, which demands further extensive research for more absolute findings.

## Introduction

Since time immemorial, mankind has used plant extracts from different medicinal plants to cure many diseases and thus relieve physical agony, playing an essential role in the world’s healthcare (Yadav et al., 2006). A wide array of phytoconstituents, called secondary metabolites, which do not appear to contribute directly to plant growth and reproduction, are responsible for the pharmacological and therapeutic effects. Thus, purification, isolation, and bioactive assessment of such secondary metabolites from medicinal and vegetative plants are widely practiced (Duraipandiyan et al., 2006).

Araceae plants (family Arum ) are commonly known as “aroids” and are distributed all over the world, and are abundant in tropical and sub-tropical regions (Ara and Hassan, 2019). *Colocasia* is one of the 27 genera of the Araceae family available in Bangladesh. It is also native to southeastern Asia and the Indian subcontinent (Wagner et al., 1990; Ara and Hassan, 2019). In Bangladesh, this genus of flowering plants is known to contain the following nine species: *C. affinis* Schott, *C. esculenta* (L.) Schott, *C. fallax* Schott, *C. gigantea* (Blume) Hook. f., *C. heterochroma* H. Li et Z.X. and Wei, *C. lihengiae* C.L. Long et K.M. Liu, *C. mannii* Hook. f., *C. oresbia* A. Hay, and *C. virosa* Kunth (Ara and Hassan, 2012). *Colocasia* leaves have demonstrated antidiabetic, antihypertensive, immunoprotective, neuroprotective, and anticarcinogenic activities. Detailed assessment of the phytochemical compounds present in various extracts of the leaves has shown the presence of active chemical compounds like anthraquinones, apigenin, catechins, cinnamic acid derivatives, vitexin, and isovitexin, which are possibly responsible for the exhibited biological properties (Gupta et al., 2019). Phytochemical extraction and structural elucidation of *Colocasia* leaves also yield notable bioactive chemical compounds, such as isoorientin, orientin, isoschaftoside, Lut-6-C-Hex-8-C-Pent, vicenin, alpha-amyrin, beta-amyrin, monoglycerol stearic acid, penduletin anthraquinones, apigenin, catechins, cinnamic acid derivatives, vitexin, and isovitexin (Gupta et al., 2019).

Pathogenic bacteria are one of the leading causes of morbidity and mortality in humans, driving pharmaceutical companies to develop numerous new antibacterial agents to address infections, which have become a global concern. Clinical microbiologists are particularly interested in plant-derived antimicrobials for two primary reasons: the potential of phytochemicals to serve as effective antimicrobial agents prescribed by healthcare professionals, and the need to increase awareness of the risks associated with the misuse of conventional antibiotics. Additionally, diarrhea is a common condition marked by frequent bouts of watery bowel movements and abdominal pain. It is a major problem in developing countries, often leading to malnutrition and even death (Workneh et al., 2024). Plant extracts have shown promise in treating diarrhea by helping the body reabsorb water, preventing electrolyte loss, and slowing gut movements (Agbor et al., 2004; Alam et al., 2020). Furthermore, inflammation manifests through a multitude of pathways and different mediators, leading to a spectrum of adverse effects like necrosis, degeneration, and various forms of exudation (Medzhitov, 2008). To alleviate pain and inflammation, analgesic medications such as opioids (like morphine and fentanyl), NSAIDs, and emerging treatments such as gabapentin, carbamazepine, and ketamine are commonly employed. Glucocorticoids exert their effects by binding to receptors, resulting in enhanced transcription of anti-inflammatory proteins (such as IL-1 antagonists) and inhibition of activated transcription factors (e.g., NF-κB) (Barnes, 1998). NSAIDs also inhibit cyclooxygenase enzymes (COX-1 and COX-2), which are responsible for synthesizing various inflammatory mediators. Despite the array of NSAIDs available, they pose significant side effects, including gastrointestinal ulceration, liver toxicity, and kidney disease, particularly with prolonged usage (Sostres et al., 2010). More specifically, COX-2 selective inhibitors run the risk of harming the heart, while non-selective inhibitors tend to wreak havoc on the gut and kidneys (Emon et al., 2021a). Hence, exploring novel phytochemicals holds promise for the development of more effective alternatives (Liu, 2007).

In addition to *in vitro* and *in vivo* studies, in today’s scientific realms, computational biology plays a pivotal role, in facilitating the generation and validation of vast amounts of data used by today’s molecular and experimental biologists. Presently, this paradigm enables the meticulous exploration and verification of drug design endeavors for nascent molecules. The adoption of computer-aided drug discovery (CADD) techniques alongside molecular docking stands as an efficacious and expedient *in silico* approach (Parmar et al., 2023; Parmar et al., 2020). Prudent molecular docking methodologies must adeptly discern the positioning of the native ligand within the three-dimensional confines of the binding site of the protein structure while also taking into account their intricate physicochemical interactions (Parmar et al., 2022; Guedes et al., 2014).

The present work was carried out for the isolation and identification of bioactive secondary metabolites from the species *C. gigantea* and *C. affinis*, along with *in vitro*, *in vivo,* and *in silico* investigation of the bioactivities of the identified compounds. Isolation and identification were carried out by chromatographic separation, followed by ^1^H-NMR analysis. Then, the identified compounds were employed for the assessment of their antibacterial antidiarrheal and analgesic potentials. Furthermore, the potential of the identified compounds for these activities was also assessed by molecular docking. An ADME/T study was done for their pharmacokinetic properties.

## Materials and methods

### Plant collection

**FIGURE 1:**
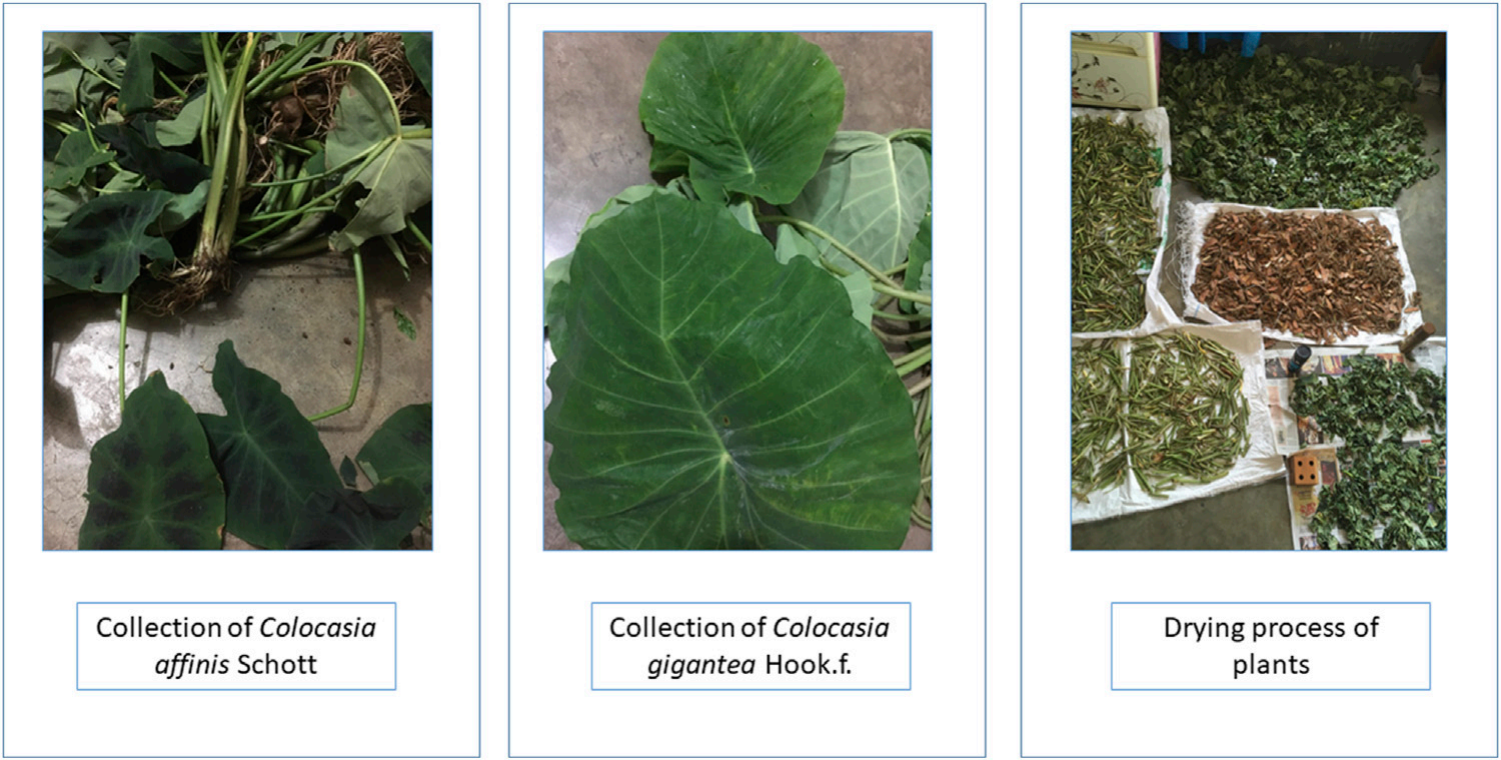
Plant parts of *C. gigantea* and *C. affinis*.

Whole plants of *C. gigantea* and *C. affinis* were collected from Bandarban and Moulovibazar, Bangladesh, in May 2019 (Figure 1). The plants were identified by the experts of Bangladesh National Herbarium, Mirpur, Dhaka, and voucher specimens (DACB; Accession numbers 57,065 and 57,066, respectively) were deposited for these collections.

### Extraction of plant material

After a proper wash, whole plants were sun-dried for several days, followed by grinding to a coarse powder using a high-capacity grinding machine. Two clean 5-L round bottom flasks were each filled with 800 g of powdered plant material soaked in 2.4 L of methanol. The containers were kept for 30 days with daily shaking and stirring. The total mixture obtained from the two containers was then filtered through a fresh cotton plug and finally through a Whatman No.1 filter paper. The volume of two filtrates was reduced by using a Buchi Rotavapor at low temperature and reduced pressure. The total weight of the crude extracts of *C. gigantea* and *C. affinis* was found to be 60.82 gm and 71.25 gm, respectively.

### Drugs and chemicals

Analytical-grade medicines and substances were employed in this investigation. Methanol and Tween-80 were bought from Merck (Darmstadt, Germany). Diclofenac sodium, loperamide, ibuprofen, azithromycin, amoxicillin, ciprofloxacin, and fluconazole were purchased from Square Pharmaceuticals Ltd., Bangladesh.

### Test microorganisms

For the antimicrobial assay, Gram-positive bacteria (*Sarcina lutea, Bacillus megaterium, Staphylococcus aureus*, *Bacillus cereus,* and *Bacillus subtilis*), Gram-negative bacteria (*Pseudomonas aeruginosa, Salmonella typhi, Salmonella paratyphi, Escherichia coli*, *Shigella dysenteriae*), and fungal strains (*Aspergillus niger*, *Saccharomyces cerevisiae*, and *Candida albicans*) were utilized, provided by the University of Dhaka, Bangladesh.

### Experimental animal models

To conduct the *in vivo* experiment, 4–5 week-old Swiss albino mice of both sexes were acquired from the Animal Resource Branch of the International Centre for Diarrheal Diseases and Research, Bangladesh (ICDDR,B). The mice were kept in standard polypropylene cages with a 12-h light-dark cycle. Other optimal conditions, including controlled room temperature of 24°C ± 2°C and relative humidity of 60%–70%, and feeding with formulated rodent food and water (*ad libitum*), were also maintained. During the experiments, all the guidelines regarding the use and care of laboratory animals, ethical rules, and regulations were implemented while designing the research and experiments. An intraperitoneal anesthetic overdose of ketamine HCl (100 mg/kg) and xylazine (7.5 mg/kg) was administered to the mouse models at the end of the experiment, followed by euthanasia. All experiments were conducted following the guidelines for the care and use of laboratory animals, which were approved by the institutional ethics committee (Zimmermann, 1983). The Animal Ethics Number for the experimental animal models of this work is 2023-01-04/SUB/A-ERC/002, indicating approval by the Animal Ethics Committee, State University of Bangladesh. This ethical certificate was issued by Prof. Dr. Mohammed Ibrahim, Chairman, Animal Ethics Committee, State University of Bangladesh (Date: 04/01/2023).

### General experimental procedures for compound isolation

**FIGURE 2:**
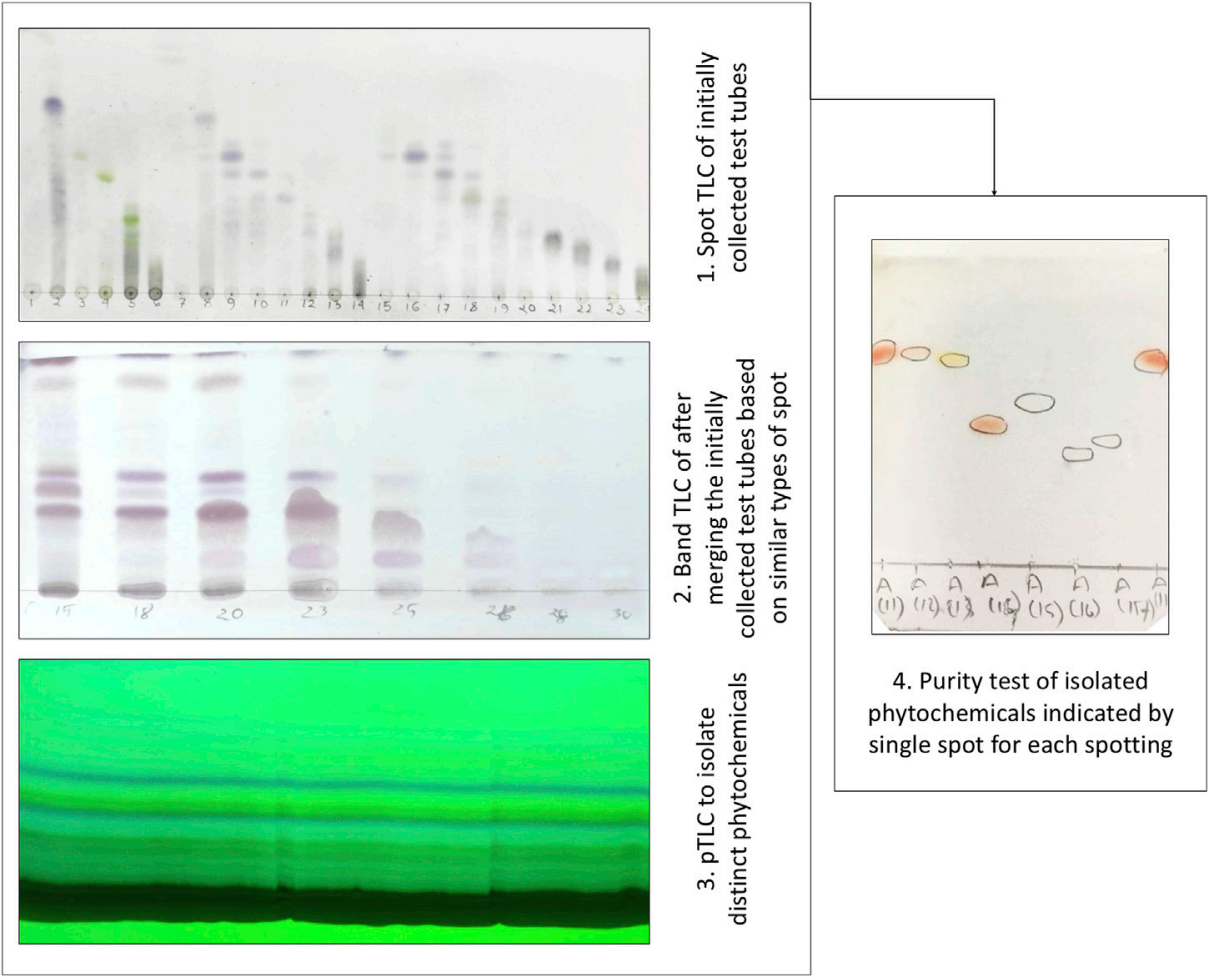
Isolation and purity testing of phytochemicals employing thin layer chromatographic (TLC) techniques.

Solvent–solvent partitioning was done by using the protocol designed by Kupchan and modified by VanWagenen et al. (1993) to avail four fractions: *n*-hexane, dichloromethane, ethyl acetate, and aqueous fraction. Gel permeation chromatography (GPC/SEC) was performed on Sephadex (LH-20) (Sigma-Aldrich) along with PTLC and TLC conducted on a silica gel 60 F_254_ on aluminum sheets with a thickness of 0.25 mm (Merck, Germany), which were observed under a UV lamp (UVGL-58, United States) at 254 nm and 365 nm. Visualization of the developed plates was done after spraying the vanillin-sulfuric acid mixture, followed by heating for 5 min at 100°C. Thus, pure compounds were isolated by the PTLC method, and the purities of the compounds were analyzed by the subsequent spot TLC method (Figure 2). The isolated compounds were then subjected to ^1^H-NMR. The ^1^H-NMR techniques were performed on a Bruker VNMRS 500 and Bruker Ascend 400 instrument using CDCl_3_ as a solvent, and the chemical shifts were documented in the δ ppm scale, keeping TMS as a reference (Ashrafi et al., 2022).

### Experimental design

#### *In vitro* tests

##### Antibacterial assay: disk diffusion test

The disk diffusion technique was used to evaluate the antibacterial properties of various fractionates of the crude extract (Huys et al., 2002). In this conventional approach, test samples were evenly distributed on nutrient agar medium that had been pre-inoculated with test bacteria using sterilized and desiccated filter paper discs (6 mm). Three commercially available antibiotic discs (azithromycin, amoxicillin, and ciprofloxacin) were considered positive standard blank discs, while fluconazole was used against fungal strains. The plates were kept for approximately 24 h upside down at a low temperature (4°C) to enable maximum diffusion of the test samples into the medium. After being flipped over, the plates were kept in an incubator set at 37°C for a whole day. Samples with antibacterial potential were deeply diluted in the medium and exhibited growth inhibition.

#### *In vivo* tests

##### Antidiarrheal bioassay: castor oil-induced diarrhea test

To assess the antidiarrheal potential of the isolated and identified compounds from *C. gigantea* and *C. affinis*, they were tested on castor oil-induced diarrheal mice. In this study, the mice were divided into twelve groups: control, positive control, and ten test groups, each containing three mice. The control group was administered with a dose of 10 mL/kg of 1% Tween 80 in a water vehicle orally. The positive control or standard group was administered with a dose of 5 mg/kg of loperamide orally (Rudra et al., 2020). Simultaneously, the two test groups were administered orally with the test compounds C1, C2, C3, and C5, and the mixture of C5 and C6 each at doses of 10 mg/kg and 20 mg/kg, respectively. One hour following the administration of the test samples, 1 mL of highly pure analytical-grade castor oil was fed to each mouse to induce diarrhea. After that, each mouse was individually placed on the box floor lined with transparent paper, and during an observation period of 5 h, the number of diarrheal feces excreted by each animal was recorded.

Throughout the treatment, each mouse was placed in an individual cage, and the floor lining was changed every hour. To evaluate the antidiarrheal activity of the test compounds, the observations of the test groups were compared against those of the control group. During the observational period of 4 h, the number of fecal stool spots was documented for each mouse. The percent inhibition of diarrhea was calculated by the following equation.

$$\%\;inhibition\;of\;defecation=\frac{Mean\;number\;of\;defecations\;by\;control-Mean\;number\;of\;defecations\;by\;test\;samples\;or\;standard}{Mean\;numbers\;of\;defecation\;by\;control}\times 100$$

##### Analgesic bioassay: acetic acid-induced writhing test

The peripheral analgesic activity of the isolated and identified compounds from *C. gigantea* and *C. affinis* was investigated in the acetic acid-induced writhing test in mice. In this study protocol, mice were divided into twelve groups: control, positive control, and ten test groups, each containing three mice. The mice in the negative control group were administered 0.1 mL of acetic acid intraperitoneally. The positive control group received standard diclofenac sodium at an oral dose of 5 mg/kg b.w. (Ahmad et al., 2010).

The two test groups were administered orally with the test compounds C1, C2, C3, C5, and the mixture of C5 and C6 each at a dose of 10 mg/kg and 20 mg/kg (b.w.; p.o.), respectively. Following the administration of the test samples, the count of writhing movements was recorded 5 min after the injection of acetic acid and documented over a period of 25 min. The percentage of writhing inhibition was subsequently calculated using the following formula:

$$\%\;Inhibition\;of\;writhing=\frac{Control\;\text{of}\;writhing\;response-Test\;\text{of}\;writhing\;response}{Control\;of\;writhing\;response}\times 100$$

##### Anti-inflammatory bioassay: formalin-induced paw-licking test

Four groups of mice, each consisting of five mice, with weights ranging from 20 g to 25 g, were subjected to the formalin-induced licking test following the protocol of Emon et al. (2021b), Alam et al. (2021a), and Sultana et al. (2022). Subcutaneous injections of 20 μL of 1% formalin solution in 0.9% saline were administered to the dorsal side of each mouse’s hind paw. The mice were transferred to a transparent observation space immediately following the injection. Then the authors measured the duration of licking time of the injected paw in rodents. Doses of 10 mg/kg and 20 mg/kg of the test compounds C1–C5 and a combination of C5 and C6 extracted from *C. affinis* and *C. gigantea* were administered orally to the groups participating in the experiment. As a standard control, a separate group was administered ibuprofen (10 mg/kg, intraperitoneally, i.p.). Additionally, a control group was administered normal saline (0.1 mL/10 g) as a baseline to compare the analgesic effects of the test extracts.

$$\%\;Inhibition\;of\;licking=\frac{Mean\;licking\;by\;the\;control\;-Mean\;licking\;by\;the\;test\;sample}{Mean\;licking\;by\;the\;control}\times 100.$$

#### *In silico* tests

##### Molecular docking

###### Software

In the *in silico* studies, the docking scores of the identified six compounds from *C. affinis* and *C. gigantea* were evaluated against the three selected biologically active target enzyme/receptor macromolecules. Various software programs, including Discovery Studio 4.5, Swiss-PDB viewer, PyRx, and PyMOL 2.3, were employed to assess the molecular interactions comprehensively (Jiko et al., 2024; Shahriar et al., 2024).

###### Ligand preparation

**FIGURE 3:**
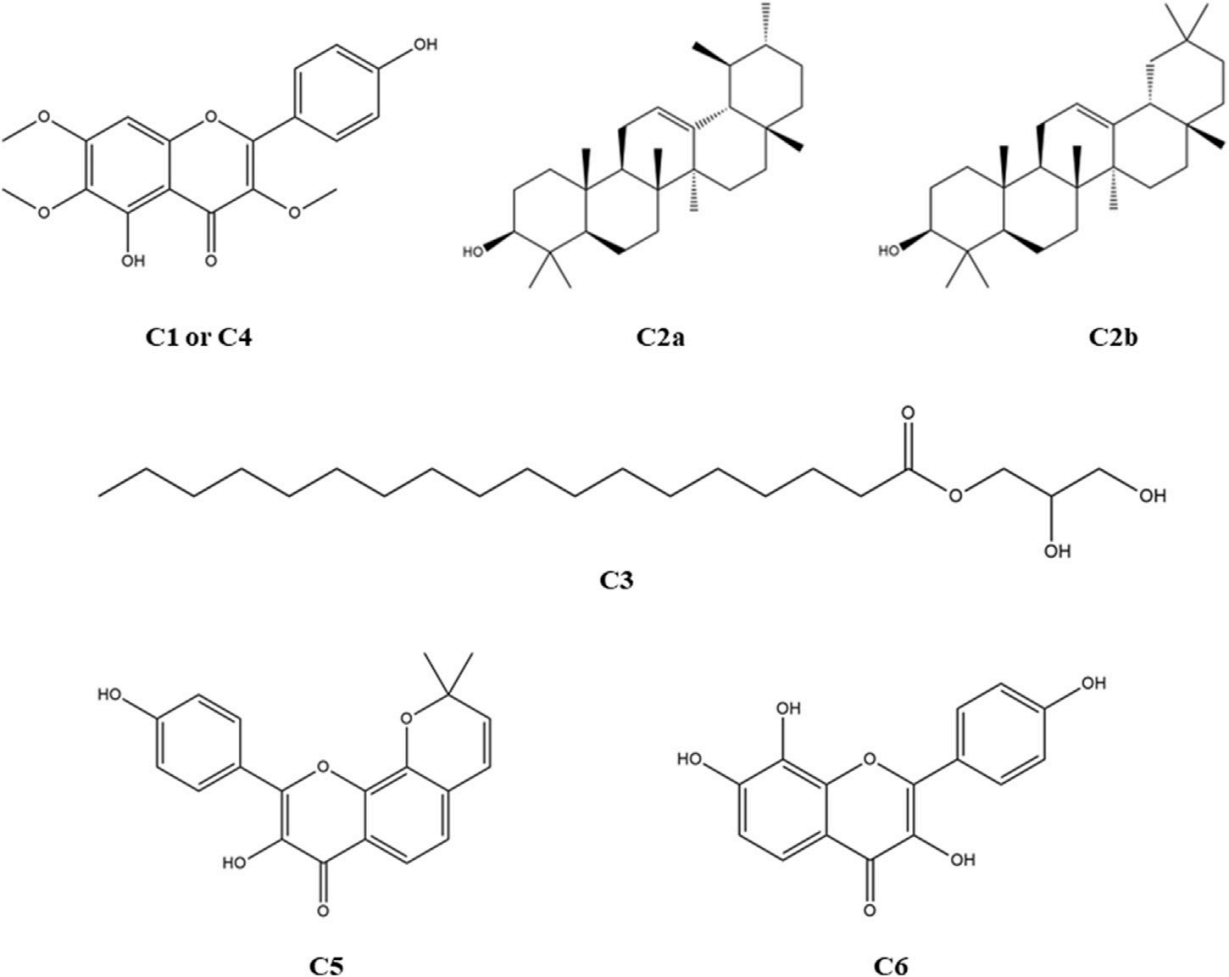
Structures of the identified phytocompounds from *C. affinis*, *C. gigantea*, and the standard drug molecules considered for the respective bioactivities.

The structures of compounds **1**–**4**, along with the standard drug molecules, were downloaded from the PubChem database (https://pubchem.ncbi.nlm.nih.gov/), and the structures of compounds **5** and **6** were drawn using ChemDraw Ultra 12.0 software. The structures of the identified compounds and standard drugs are presented in Figure 3. The ligands were downloaded in 3D SDF format and serially loaded into Discovery Studio 4.5. To improve the docking accuracy, the semiempirical technique was used to optimize all the phytoconstituents (Bikadi and Hazai, 2009).

###### Target protein preparation

The target macromolecules were obtained as 3D crystal structures from the RCBS Protein Data Bank (https://www.rcsb.org/structure) in the PDB format. For antibacterial, antidiarrheal, and peripheral analgesic activity assessment, dihydrofolate reductase (DHFR) enzyme [PDB ID: 4M6J] (Khatun et al., 2021), kappa opioid receptor (KOR) [PDB ID: 6VI4] (Alam et al., 2021b), and cyclooxygenase-2 (COX-2) enzyme [PDB ID: 1CX2] (Muhammad et al., 2015) were employed. After that, all water molecules and heteroatoms were removed from the proteins using Discovery Studio 2021, and Swiss-PDB Viewer’s energy minimization tool was used to optimize biomolecules by arranging nonpolar hydrogen atoms.

###### Ligand–protein interaction

To gain insight into how molecules interact at the molecular level, the potential binding patterns and binding affinities were predicted through a computer-aided ligand–protein interaction diagram. The software PyRx was employed for the molecular drug–protein binding procedure. A careful selection of particular amino acids, along with their corresponding IDs, was undertaken from the scientific literature for each enzyme/receptor individually, aiming for precise target docking (Khatun et al., 2021; Alam et al., 2021b; Muhammad et al., 2015). Subsequently, the protein was prepared by loading and formatting as the necessary macromolecule, guaranteeing specific ligand binding to the intended targets.

To enhance the docking process with the chosen macromolecules, ligand SD files were brought in and transformed into pdbqt format using the Open Babel tool within the PyRx software. Grid mapping identified the active amino sites within specific grid boxes, following the predetermined center and dimensional axes outlined in Table 1. During this stage, basic supportive default functions remained unchanged (Muhammad et al., 2015). The last step entailed interpreting the results and employing BIOVIA Discovery Studio version 4.5 to forecast the optimal 2D and 3D models.

**TABLE 1:** Target site selection and grid mapping of target receptors.

Receptor	Standard	Target binding sites	References	Grid box	
DHFR (4M6J)	Ciprofloxacin	Ala 9, Ile 16, Lys 54, Lys 55, Thr 56, Leu 75, Ser 76, Arg 77, Glu 78, Arg 91, Ser 92, Leu 93, Gly 117, Ser 118, Ser 119, and Val 120	Khatun et al. (2021)	Center	x = 3.20179912784
					y = −3.79824570941
					z = −18.5444224326
				Dimension	x = 25.2409863514
					y = 30.9244686771
					z = 28.5216876816
KOR (6VI4)	Loperamide	Leu 103, Leu 107, Ser 136, Ile 137, Try 140, Ile 180, Trp 183, Leu 184, Ser 187, Ile 191, Leu 192 Ile 194, and Val 195	Alam et al. (2021a)	Center	x = 53.9115751709
					y = −50.1152995766
					z = −16.2408511962
				Dimension	x = 18.2799964783
					y = 30.7770137609
					z = 20.3958600148
COX-2 (1CX2)	Diclofenac sodium	His 90, Gln 192, Val 349, Leu 352, Ser 353, Tyr 355, Tyr 385, Ala 516, Phe 518, Val 523, Ala 527, and Ser 530	Muhammad et al. (2015)	Center	x = 23.2515878129
					y = 21.7831246177
					z = 16.1794397334
				Dimension	x = 28.7572839296
					y = 22.8801400278
					z = 30.7900966105

###### ADME/T study

For the evaluation of pharmacokinetic parameters like absorption, distribution, metabolism, excretion, and toxicity (ADME/T), pkCSM (http://structure.bioc.cam.ac.uk/Pkcsm) was employed (Pires et al., 2015). Concurrently, the Swiss ADME (http://www.swissadme.ch), which predicts drug-likeness based on Lipinski’s rules and pharmacokinetic parameters (Daina et al., 2017; Shompa et al., 2024), was used to determine the drug-likeness and bioavailability score of selected compounds.

#### Statistical analysis

The statistical analysis was done through the presentation of mean values accompanied by the standard error of the mean (SEM). These values were meticulously compared to those of the control group and discerned to be statistically significant (***p < 0.001, **p < 0.01, and *p < 0.05), following a rigorous one-way analysis of variance (ANOVA) supplemented by Dunnett’s test. All statistical manipulations were conducted utilizing GraphPad Prism Version 5.2 (San Diego, CA).

## Results

### Identification of compounds

A total of six compound structures, shown in Figure 3, were isolated and identified from the *Colocasia gigantea* and *Colocasia affinis.*

**FIGURE 4:**
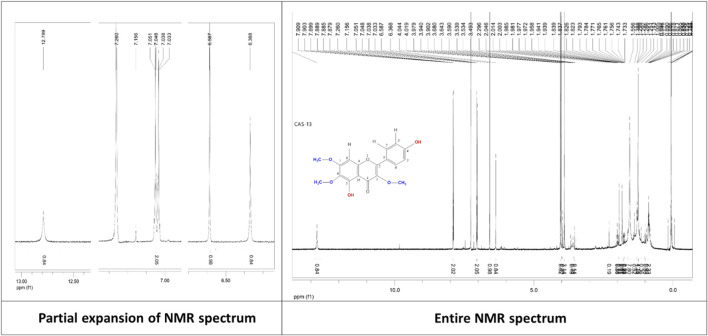
^1^H-NMR spectrum of the compound penduletin isolated from *C. gigantea* and *C. affinis*.

**Penduletin (C1 or C4):** Pale yellow crystals, 1^H^ NMR (500 MHz, CDCl_3_): δH 6.59 (1H, s, H-8), 7.90 (1H, d, *J* = 9.0 Hz, H-2′), 7.04 (1H, d, *J* = 9.0 Hz, H-3′), 7.04 (1H, d, *J* = 9.0 Hz, H-5′), 7.90 (1H, d, *J* = 9.0 Hz, H-6′), 3.90 (3H, s, 3-OCH3), 4.02 (3H, s, 6-OCH3), 4.04 (3H, s, 7-OCH3), 12.79 (1H, s, 5-OH), 6.37 (1H, s, 4′-OH). The corresponding ^1^H-NMR spectrum is depicted in Figure 4.

**FIGURE 5:**
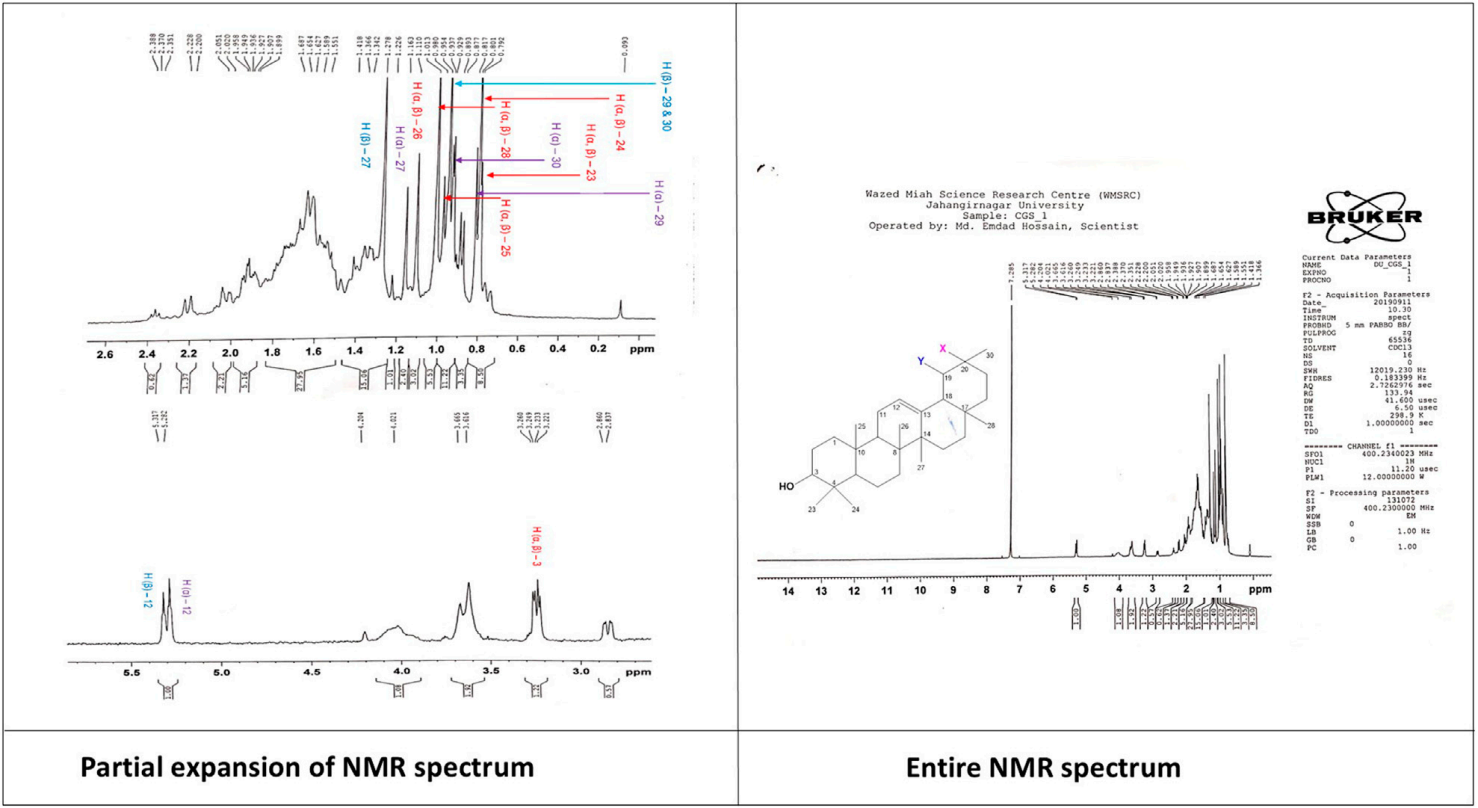
^1^H-NMR spectrum of the compound mixture of α-amyrin and β-amyrin isolated from *C. gigantea*.

**A mixture of α-amyrin and β-amyrin (C2) α-Amyrin (C2a):** White powder, ^1^H NMR (400 MHz, CDCl_3_): δ_H_ 5.28 (1H, t, H-12), 3.24 (1H, dd, *J* = 4.4, 4.8 Hz, H-3), 1.16 (3H, s, H-27), 1.11 (6H, s, H-26), 1.01 (6H,s, H-28), 0.98 (6H, s, H-25), 0.93 (3H, d, *J* = 3.2 Hz, H-30), 0.82 (3H, d, *J* = 3.2 Hz, H-29), 0.80 (s, 6H, H-23), 0.79 (s, 6H, H-24). The corresponding ^1^H-NMR spectrum is depicted in Figure 5.

**β-Amyrin (C2b):** White powder, ^1^H NMR (400 MHz, CDCl_3_): δ_H_ 5.31 (t, 1H, H-12), 3.24 (dd, 2H, *J =* 4.4, 4.8 Hz, H-3), 1.28 (s, 3H, H-27), 1.11 (s, 6H, H-26), 1.01 (s, 6H, H-28), 0.98 (s, 6H, H-25), 0.95 (s, 6H, H-29,30) 0.80 (s, 6H, H-23), 0.79 (s, 6H, H-24). The corresponding ^1^H-NMR spectrum is depicted in Figure 5.

**FIGURE 6:**
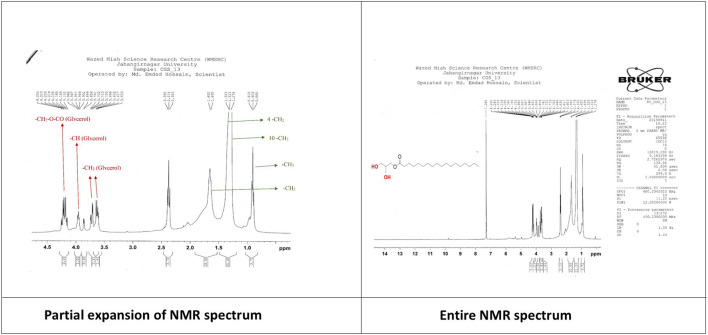
^1^H-NMR spectrum of the compound monoglyceride of stearic acid isolated from *C. gigantea*.

**Monoglyceride of stearic acid (C3):** Colorless crystals, ^1^H NMR (400 MHz, CDCl_3_); **Glycerol backbone:** -CH_2_-O-CO: 4.195 (2H, dd, *J* = 13.6, 5.6 Hz), -CH: 3.957 (1H, m), -CH_2_: 3.732 (2H, dd, *J* = 11.6, 4.0 Hz), 3.617 (1H, dd, *J* = 11.6, 5.6 Hz). **Stearic acid part:** -CH_2_-CO-O: 2.37 (t, 2H), -CH_2_: 1.64 (d, 2H), 4-CH_2_: 1.31 (d, 8H), 10-CH_2_: 1.25 (s, 20H), -CH3: 0.90 (t, 3H). The corresponding ^1^H-NMR spectrum is depicted in Figure 6.

**FIGURE 7:**
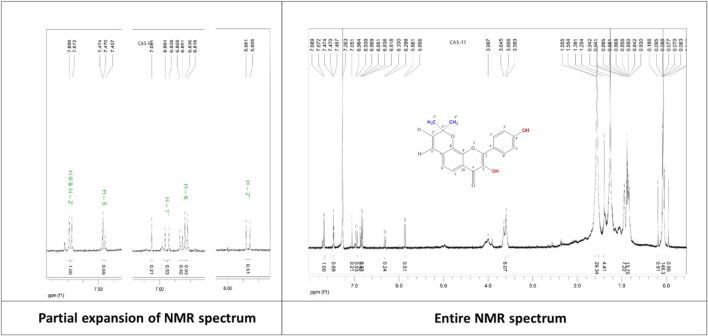
^1^H-NMR spectrum of the compound 7,8-(3″,3″-dimethyl-pyrano)-4′-hydroxy flavonol isolated from *C. affinis*.

**7,8 -(3′′,3′′-dimethyl-pyrano)-4′-hydroxy flavonol (C5):** White powder, ^1^H NMR (500 MHz, CDCl_3_): δ_H_ 7.46 (1H, d, *J* = 8.5 Hz, H-5), 6.86 (1H, d, *J* = 8.5 Hz, H-6), 7.68 (1H, d, *J* = 8.5 Hz, H-2′), 7.68 (1H, d, *J* = 8.5 Hz, H-6′), 6.96 (2H, d, *J* = 12.5 Hz, H-1″), 6.84 (1H, d, *J* = 9.0 Hz, H-3′), 6.84 (1H, d, *J* = 9.0 Hz, H-5′), 5.87 (1H, d, *J* = 12.5 Hz, H-2″), 1.55 (6H, s, H-4″, H-5″). The corresponding ^1^H-NMR spectrum is depicted in Figure 7.

**FIGURE 8:**
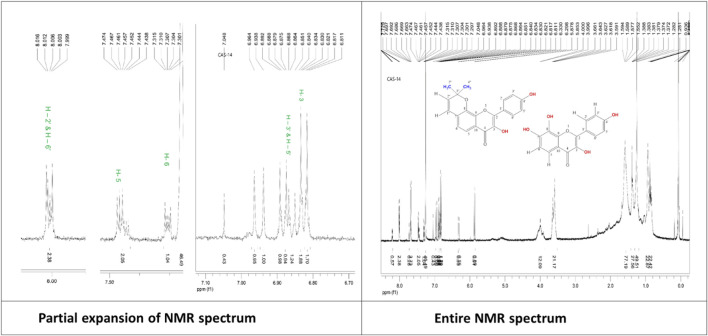
^1^H-NMR spectrum of the compound mixture of 7,8-(3″,3″-dimethyl-pyrano)-4′-hydroxy flavonol and 4',7,8-trihydroxy flavonol isolated from *C. affinis*.

**Mixture of 7,8-(3′′,3′′-dimethyl-pyrano)-4′-hydroxy flavonol (C5) and 4′,7,8-trihydroxy flavonol (C6):** White powder, ^1^H NMR (500 MHz, CDCl_3_): δ_H_ 6.81 (1H, s, H-3), 7.46 (1H, d, *J* =9.0 Hz, H-5), 7.30 (1H, d, *J* = 9.0 Hz, H-6), 8.00 (1H, d, *J* = 8.5 Hz, H-2′), 6.88 (1H, d, *J* = 8.5 Hz, H-3′), 6.88 (1H, d, *J* = 8.5 Hz, H-5′), 8.00 (1H, d, *J* = 8.5 Hz, H-6′). The corresponding ^1^H-NMR spectrum is depicted in Figure 8.

### *In vitro* testing

#### Effect of the identified test compounds on the disc diffusion assay

The antibacterial activity of all the partitions was tested against five strains of gram-positive and gram-negative bacteria and three strains of fungi. As a reference standard, azithromycin, amoxicillin, ciprofloxacin, and fluconazole were taken to test the respective antimicrobial activity. The zone of inhibition (ZOI) of the test samples ranged from 6 mm to 20 mm and is summarized in Table 2. C1/C4, C2, C3, and C5 showed considerable antibacterial activity, whereas C1/C4 and C3 exhibited promising antifungal attributes. As per ZOI, the fractionated extracts exerted notable antimicrobial activities against *B. cereus*, *B. megaterium*, *B. subtilis*, *S. aureus*, *S. lutea*, *E. coli*, *P. aeruginosa,* and *S. dysenteriae* and relatively lower ZOI against *S. paratyphi, S. typhi, A. niger, C. albicans*, and *S. cerevisiae.* In addition, all the standard drugs exhibited the expected pronounced ZOIs against all the tested strains, ranging from 27 mm to 45 mm.

**TABLE 2:** Antibacterial activity of the isolated compounds from *C. gigantea* and *C. affinis* against Gram-positive and Gram-negative bacteria.

Test microorganisms	Zone of inhibition (mm)							
	Azithromycin (30 μg/disc)	Amoxicillin (30 μg/disc)	Ciprofloxacin (30 μg/disc)	C1/C4 (100 μg/disc)	C2 (100 μg/disc)	C3 (100 μg/disc)	C5 (100 μg/disc)	Mixture of C5 and C6 (100 μg/disc)
Gram-positive bacteria								
*Bacillus cereus*	37	35	31	15	12	8	11	—
*Bacillus megaterium*	35	32	30	14	11	5	12	—
*Bacillus subtilis*	34	28	32	9	10	6	7	—
*Staphylococcus aureus*	41	39	33	18	17	14	8	8
*Sarcina lutea*	37	35	29	16	13	15	11	—
Gram-negative bacteria								
*Escherichia coli*	38	36	34	17	14	11	7	—
*Pseudomonas aeruginosa*	41	37	38	20	13	10	8	6
*Salmonella paratyphi*	30	31	27	15	8	7	6	—
*Salmonella typhi*	39	31	36	18	16	8	—	8
*Shigella dysenteriae*	37	32	33	17	15	12	6	—
Fungi								

Test Microorganisms	Fluconazole (30 μg/disc)			C1/C4 (100 μg/disc)	C2 (100 μg/disc)	C3 (100 μg/disc)	C5 (100 μg/disc)	Mixture of C5 and C6 (100 μg/disc)
*Aspergillus niger*	45			12	5	9	6	—
*Candida albicans*	38			—	—	—	—	—
*Saccharomyces cerevisiae*	41			9	—	7	—	—

### *In vivo* study

#### Effect of the identified test compounds on castor oil-induced diarrhea

Compounds C2, C5, and the mixture of C5 and C6, each at doses of 10 mg/kg and 20 mg/kg, and compound C1 at a dose of 20 mg/kg exhibited significant (*p* < 0.05, *p* < 0.01) reduction in the number of feces (Table 3). In terms of wet feces number, C2, C5, and the mixture of C5 and C6 demonstrated percentages of diarrhea inhibition of 29.63%, 37.04%, and 25.93%, respectively, at the 10 mg/kg dose, while at the 20 mg/kg dose, C1, C2, C5, and the mixture of C5 and C6 exhibited reductions of 33.33%, 40.74%, 44.44%, and 37.04%, respectively. The value of the standard loperamide was 77.78%.

**TABLE 3:** Antidiarrheal and analgesic activities of isolated compounds from *C. gigantea* and *C. affinis,* respectively, on castor oil-induced diarrhea and acetic acid-induced writhing test in mice.

Animal group with corresponding doses (ml/kg or mg/kg, b.w.; p.o.)	Number of diarrheal feces (Mean ± SEM)	% reduction in diarrhea	Number of writhing episodes (Mean ± SEM)	% reduction in writhing
CTL	9 ± 0.58	—	15.33 **±** 0.33	—
STD (loperamide/diclofenac sodium)	2 ± 0.58***	77.78	3.67 **±** 0.67***	76.09
Compound 1 or 4 (10 mg/kg b.w.)	7.33 ± 0.88	18.52	10.33 **±** 0.67**	32.61
Compound 1 or 4 (20 mg/kg b.w.)	6 ± 0.58*	33.33	6.67 **±** 0.68**	56.52
Compound 2 (10 mg/kg b.w.)	6.33 ± 0.67*	29.63	9.33 **±** 0.33***	39.13
Compound 2 (20 mg/kg b.w.)	5.33 ± 0.33**	40.74	6.67 **±** 0.33***	56.52
Compound 3 (10 mg/kg b.w.)	7.67 ± 0.33	14.81	11.67 **±** 0.33**	23.91
Compound 3 (20 mg/kg b.w.)	7 ± 0.001	22.22	11.33 **±** 0.33**	26.09
Compound 5 (10 mg/kg b.w.)	5.67 ± 0.33*	37.04	11 ± 0.58**	28.26
Compound 5 (20 mg/kg b.w.)	5 ± 0.58**	44.44	8.33 ± 0.88**	45.65
Mixture of compounds 5 and 6 (10 mg/kg b.w.)	6.67 ± 0.33*	25.93	10.67 **±** 0.33***	30.43
Mixture of compounds 5 and 6 (20 mg/kg b.w.)	5.67 ± 0.33*	37.04	7.67 **±** 0.33***	50.0

Values are expressed as mean ± SEM (n = 3); CTL, negative control; STD, positive control; ***p < 0.001, **p < 0.01, *p < 0.05 compared to negative control.

#### Effect of the identified test compounds on acetic acid-induced writhing in the mice model

All the test compounds C1, C2, C3, C5, and the mixture of C5 and C6 each at 10 and 20 mg/kg doses exhibited significant (*p* < 0.01, *p* < 0.001) analgesia with a considerable percentage reduction of acetic acid-induced writhing compared to the standard diclofenac sodium (Table 3). Among them, C1 and C2 exhibited the highest percentage reduction of writhing with 56.52% at the 20 mg/kg dose when compared to the standard of 76.09%.

#### Effect of the identified test compounds on formalin-induced licking in the mice model

The administration of test compounds C1/C4, C2, C3, and C5, and the combination of C5 and C6 at doses of 10 mg/kg and 20 mg/kg resulted in significant analgesic effects, as evidenced by a marked reduction in formalin-induced paw licking behavior. The observed anti-inflammatory effect was statistically significant (*p* < 0.01, *p* < 0.001) **(** Table 4), demonstrating a marked reduction in formalin-induced writhing relative to the control group receiving ibuprofen. C5 and C2 demonstrated the highest anti-inflammatory efficacy among the compounds evaluated, resulting in a 64.50% and 68.15% reduction in licking during the early phase and a 66.66% and 52.06% reduction during the later phase, respectively, at a dosage of 20 mg/kg. On the other hand, the standard drug ibuprofen led to a decrease of 73.54% and 74.68% in early and late phase subsequently. The findings indicate that compounds C5 and C2 demonstrate substantial potential for development as anti-inflammatory agents, primarily due to their marked reduction in pain responses in the examined mice.

**TABLE 4:** Anti-inflammatory effect of the isolated compounds from *C. gigantea* and *C. affinis* on the formalin-induced mouse model.

Animal group with respective doses (ml/kg or mg/kg, b.w; p.o)	Time of licking in seconds (Ealy Phase; 0–5 min)	Time of licking in seconds (Late Phase; 15–30 min)
CTL	60.38 ± 2.20	73.68 ± 1.34
STD (Ibuprofen, 10 mg/kg, b.w)	15.98 ± 1.62***	18.65 ± 2.52***
Compound 1 or 4 (10 mg/kg, b.w.)	32.54 ± 3.12***	38.87 ± 1.09***
Compound 1 or 4 (20 mg/kg, b.w.)	31.38 ± 1.12***	27.61 ± 1.02***
Compound 2 (10 mg/kg, b.w.)	44.32 ± 1.52***	51.49 ± 0.71***
Compound 2 (20 mg/kg, b.w.)	19.23 ± 3.34***	35.32 ± 1.77***
Compound 3 (10 mg/kg, b.w.)	38.04 ± 4.19**	45.68 ± 1.95***
Compound 3 (20 mg/kg, b.w.)	28.43 ± 2.38***	30.56 ± 2.16***
Compound 5 (10 mg/kg, b.w.)	40.38 ± 1.51***	53.29 ± 1.43***
Compound 5 (20 mg/kg, b.w.)	21.43 ± 0.53***	24.56 ± 0.98***
Mixture of compound 5 and 6 (10 mg/kg, b.w.)	44.28 ± 2.20**	56.21 ± 2.40**
Mixture of compound 5 and 6 (20 mg/kg, b.w.)	30.44 ± 1.05***	36.43 ± 4.81**

Values are expressed as mean ± SEM (n = 5); CTL, negative control; STD, positive control; ***p < 0.001, **p < 0.01, *p < 0.05 compared to negative control.

### *In silico* study

#### Molecular docking

**FIGURE 9:**
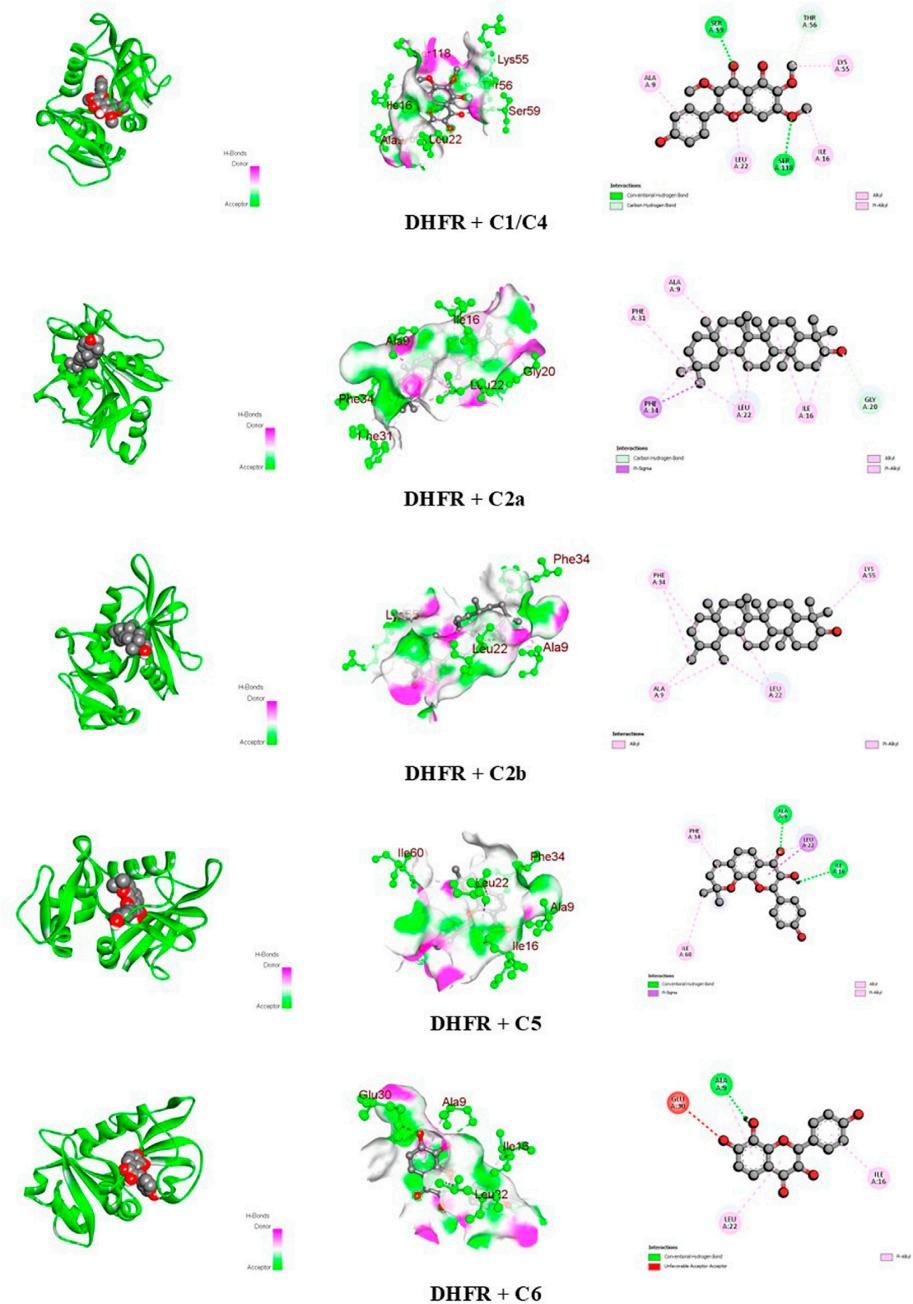
Graphical representation of the molecular interactions of the isolated phytocompounds with the DHFR enzyme with 3D and 2D visualization.

**FIGURE 10:**
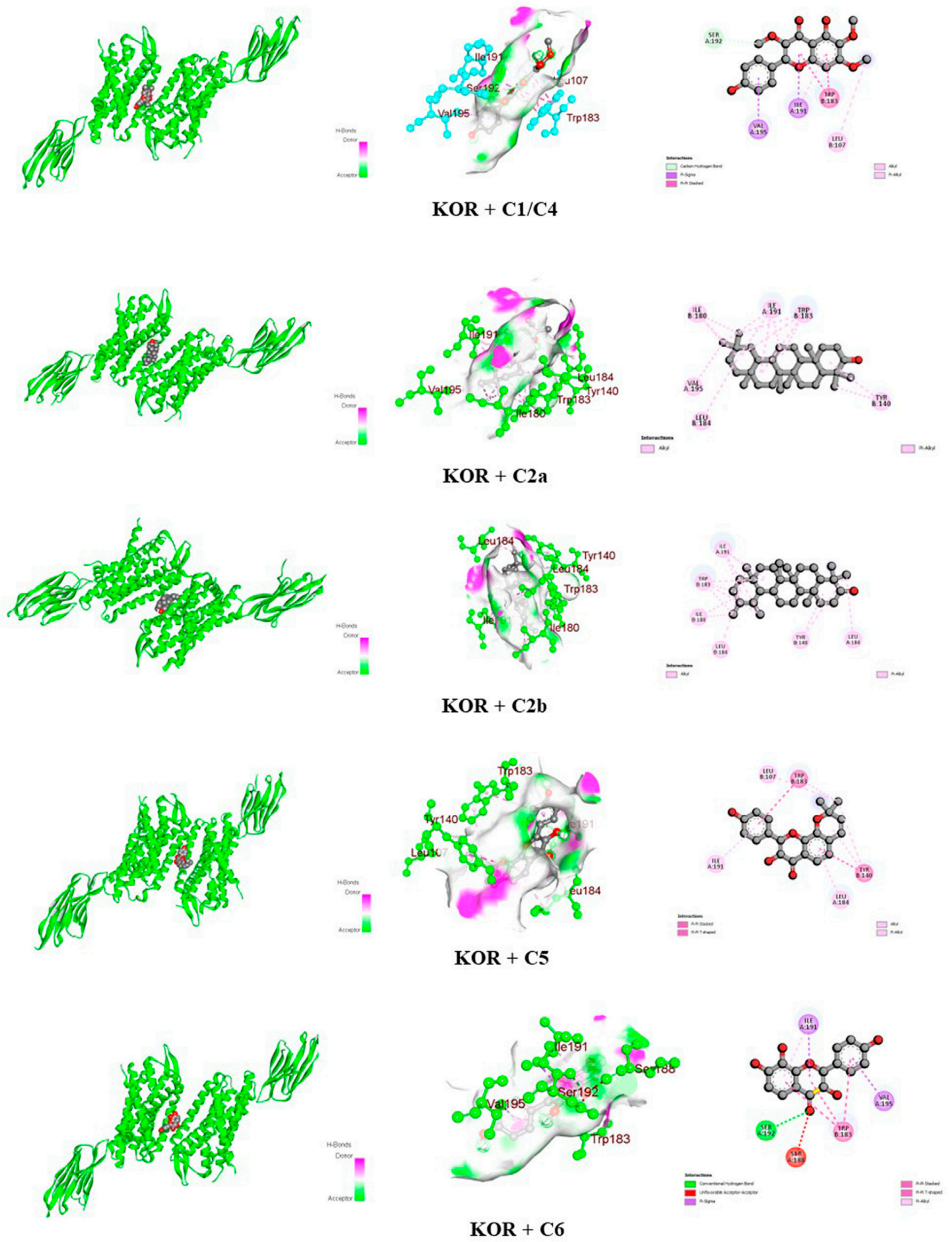
Graphical 3D and 2D visualization of the molecular interactions of the isolated phytocompounds with the KOR receptor.

**FIGURE 11:**
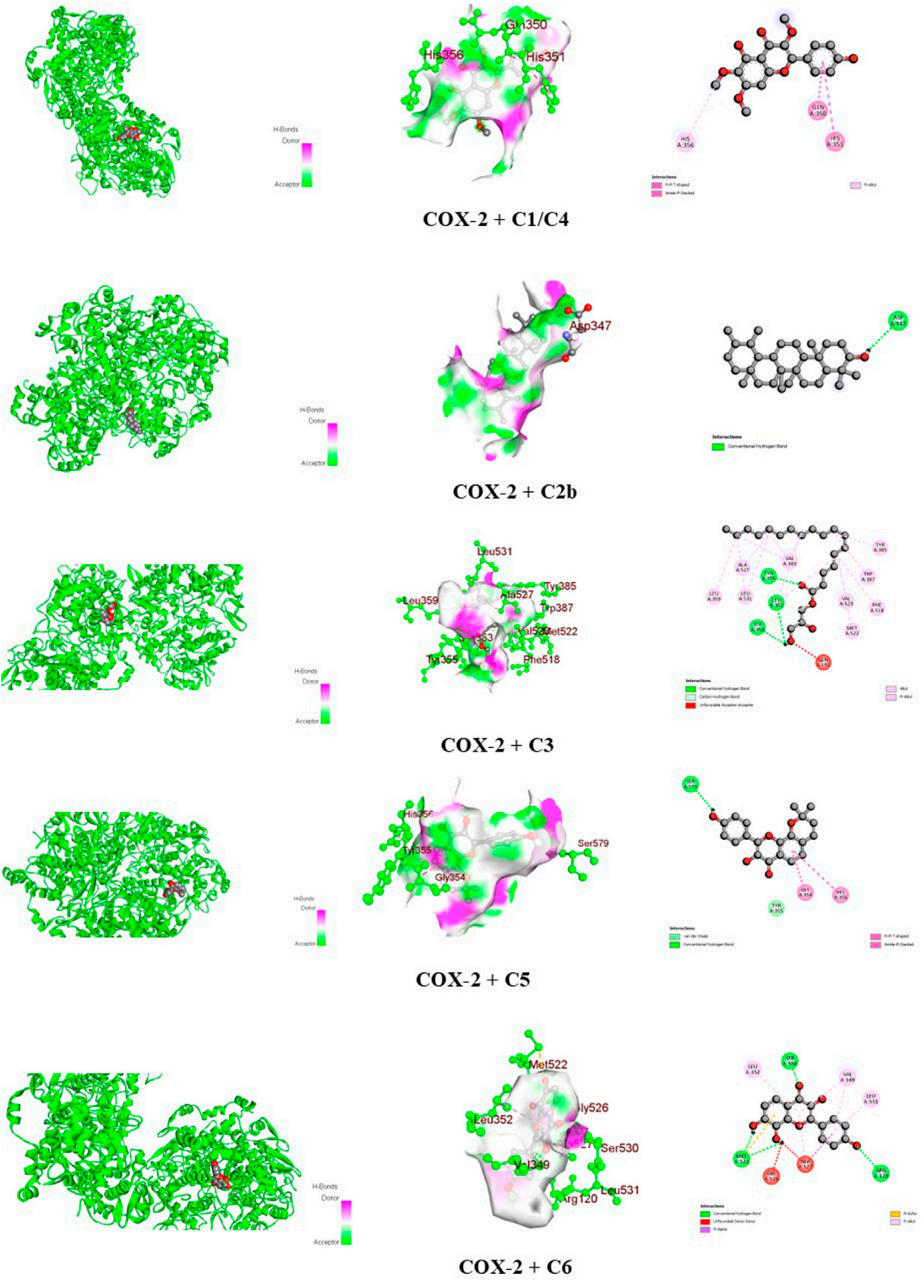
Graphical 3D and 2D visualization of the molecular interactions of the isolated phytocompounds with the COX-2 enzyme.

**FIGURE 12:**
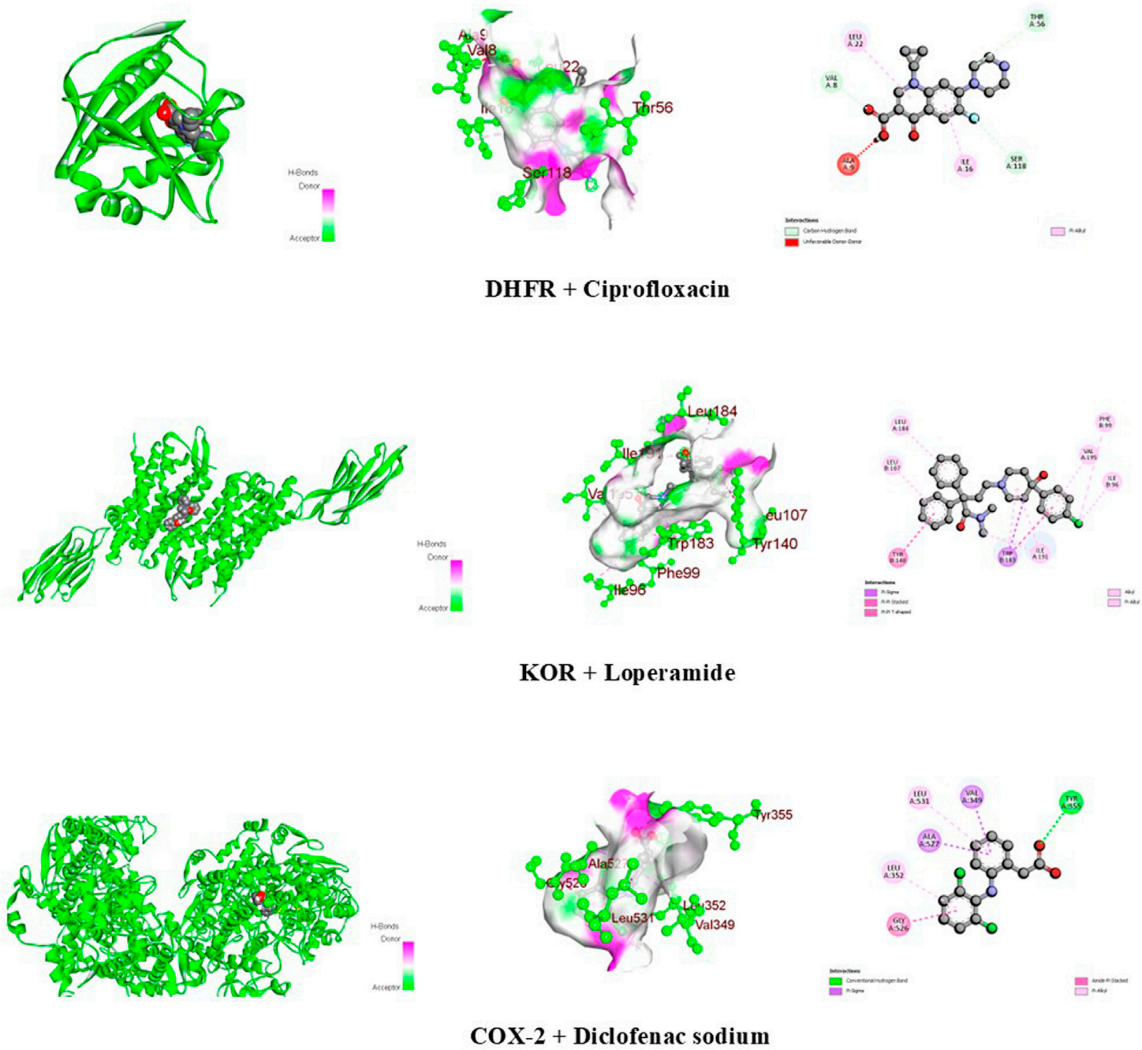
Graphical 3D and 2D visualization of the molecular interactions of the standard drug molecules with their respective enzymes.

The docking scores of the identified compounds from the methanolic extracts of *C. affinis* and *C. gigantea* to selected targets are depicted in Table 5. For the enzyme DHFR, **C5** showed the highest docking score of −9.1 kcal/mol, followed by **C2a**, **C2b,** and **C1/C4** with promising docking scores of −8.8 kcal/mol, −8.8 kcal/mol, and −7.9 kcal/mol, respectively, when compared to the standard drug ciprofloxacin with a docking score of −8.1 kcal/mol. The 3D and 2D graphical representations of the molecular interactions of these compounds and the standard drug ciprofloxacin with the DHFR enzyme are depicted in Figures 9, 12, respectively. In the case of the KOR receptor, the standard drug loperamide showed a docking score of −9.1 kcal/mol, while the test compound **C2b** showed the highest docking score of −10.5 kcal/mol. The other compounds also showed significant scores of −9.7 kcal/mol **(C5)**, −9.4 kcal/mol **(C2a)**, −8 kcal/mol **(C6)**, and −7.7 kcal/mol **(C1/C4)**. The 3D and 2D graphical representations of the molecular interactions of these compounds and the standard loperamide with the KOR receptor are depicted in Figures 10, 12. Additionally, with the COX-2 enzyme, the compound C2b showed a promising docking score of −8.7 kcal/mol, along with other notable docking scores: −7.3 kcal/mol **(C6)**, −7.2 kcal/mol **(C5)**, and −7 kcal/mol **(C1 and C3)**. The docking score of the standard drug diclofenac sodium to the COX-2 enzyme was −7.8 kcal/mol. The 3D and 2D graphical representations of the molecular interactions of these compounds and the standard diclofenac sodium with COX-2 enzyme are depicted in Figures 11, 12. The corresponding binding interactions and the binding sites of highly active compounds against the targets, including DHFR, KOR, and COX-2, are presented in Table 6.

**TABLE 5:** Binding affinities of the identified compounds from *C. gigantea* and *C. affinis* and standards against three macromolecules, namely, DHFR, KOR, and COX-2, representing antibacterial, antidiarrheal, and peripheral analgesic activities, respectively.

Compound code	Compound name	Molecular formula	Molecular weight (g/mol)	Binding affinities (kcal/mol)		
				Antibacterial	Antidiarrheal	Analgesic
				DHFR (4M6J)	KOR (6VI4)	COX-2 (1CX2)
C1/C4	Penduletin	C_18_H_16_O_7_	344.3	−7.9	−7.7	−7
C2a	α-Amyrin	C_30_H_50_O	426.7	−8.8	−9.4	−5.3
C2b	β-Amyrin	C_30_H_50_O	426.7	−8.8	−10.5	−8.7
C3	Monoglyceride of stearic acid	C_21_H_42_O_4_	358.6	−5.4	−6.3	−7
C5	7,8-(3″,3″-Dimethyl-pyrano)-4′-hydroxy flavonol	C_20_H_16_O_5_	336.34	−9.1	−9.7	−7.2
C6	4′,7,8-Trihydroxy flavonol	C_15_H_10_O_6_	286.24	−8.1	−8	−7.3
Standards	Ciprofloxacin		331.34	−8.1	—	—
	Loperamide		477	—	−9.1	—
	Diclofenac sodium		318.1	—	—	−7.8

**TABLE 6:** Bonds and binding sites of the identified best-binding compounds from *C. gigantea* and *C. affinis* against different targets, including DHFR, KOR, and COX-2.

Receptor	Compound	Docking score (kcal/mol)	Bond type	Amino acids
DHFR (4M6J)	C1/C4	−7.9	Conventional hydrogen	Ser 59 and Ser 118
			Carbon-hydrogen	Thr 56
			Alkyl	Ile 16 and Lys 55
			Pi-alkyl	Ala 9 and Leu 22
	C2a	−8.8	Carbon-hydrogen bond	Gly 20
			Pi-sigma	Phe 34
			Alkyl	Ala 9, Ile 16, Leu 22, and Phe 31
	C2b	−8.8	Alkyl	Ala 9, Leu 22, Phe 34, and Lys 55
	C5	−9.1	Conventional hydrogen	Ala 9 and Ile 16
			Pi-sigma	Leu 22
			Alkyl	Ile 60
			Pi-alkyl	Phe 34
	C6	−8.1	Conventional hydrogen	Ala 9
			Unfavorable acceptor–acceptor	Glu 30
			Pi-alkyl	Ala 9, Ile 16, and Leu 22
	Ciprofloxacin	−8.1	Carbon-hydrogen	Val 8, Thr 56, and Ser 118
			Unfavorable donor-donor	Ala 9
			Pi-alkyl	Ile 16 and Leu 22
KOR (6VI4)	C1/C4	−7.7	Carbon-hydrogen	Ser 192
			Pi-sigma	Ile 191 and Val 195
			Pi-pi stacked	Trp 183
			Alkyl	Leu 107
			Pi-alkyl	Ile 191
	C2a	−9.4	Alkyl	Tyr 140, Ile 180, Trp 183, Leu 184, Ile 191, and Val 195
	C2b	−10.5	Alkyl	Tyr 140, Ile 180, Trp 183, Leu 184, and Ile 191
	C5	−9.7	Pi-pi stacked	Tyr 140 and Trp 183
			Alkyl	Leu 107, Leu 184, and Ile 191
	C6	−8	Conventional hydrogen	Ser 192
			Unfavorable acceptor-acceptor	Ser 188
			Pi-sigma	Ile 191 and Val 195
			Pi-pi stacked	Trp 183
			Pi-alkyl	Ile 191
	Loperamide	−9.1	Pi-sigma	Trp 183
			Pi-pi stacked	Tyr 140 and Trp 183
			Alkyl	Ile 96, Phe 99, Leu 107, Leu 184, Trp 183, Ile 191, and Val 195
COX-2 (1CX2)	C1/C4	−7	Pi-pi T-shaped	Gln 350 and His 351
			Pi-alkyl	His 356
	C2b	−8.7	Conventional hydrogen	Asp 347
	C3	−7	Conventional hydrogen	Leu 352, Ser 353, and Tyr 355
			Carbon-hydrogen	Leu 352 and Ser 353
			Unfavorable acceptor-acceptor	Gln 192
			Alkyl	Val 349, Leu 359, Tyr 385, Trp 387, Phe 518, Met 522, Val 523, Ala 527, and Leu 531
	C5	−7.2	Van der Waals	Tyr 355
			Conventional hydrogen	Ser 579
			Pi-pi T-shaped	Gly 354 and His 356
	C6	−7.3	Conventional hydrogen	Arg 120, Met 522, and Ser 530
			Unfavorable donor-donor	Gly 526 and Ala 527
			Pi-sigma	Ala 527
			Pi-sulfur	Met 522
			Pi-alkyl	Val 349, Leu 352, Ala 527, and Leu 531
	Diclofenac sodium	−7.8	Conventional hydrogen	Tyr 355
			Pi-sigma	Val 349 and Ala 527
			Amide-pi stacked	Gly 526
			Pi-alkyl	Leu 352 and Leu 531

#### ADME/T study

The *in silico* study also analyzed the ADME/T parameters of the identified compounds. The bioavailability score and Lipinski’s rule of five data were also considered for the assessment of drug-likeness of the compounds, which are demonstrated in Table 7.

**TABLE 7:** ADME/T study of the identified best-binding compounds from *C. gigantea* and *C. affinis* against DHFR, KOR, and COX-2 macromolecules.

Property	Model name (Unit)	C1/C4	C2a	C2b	C3	C5	C6
Absorption	Water solubility (log mol/L)	−3.386	−6.499	−6.531	−6.044	−3.975	−3.114
	CaCo_2_ permeability (log Papp in 10–6 cm/s)	−0.045	1.227	1.226	0.362	0.91	0.389
	Intestinal absorption (human) (% absorbed)	88.746	94.062	93.733	90.234	94.821	84.522
	Skin permeability (log Kp)	−2.741	−2.814	−2.811	−2.814	−2.744	−2.735
	P-glycoprotein substrate	Yes	No	No	No	Yes	Yes
	P-glycoprotein I inhibitor	No	Yes	Yes	Yes	No	No
	P-glycoprotein II inhibitor	Yes	Yes	Yes	Yes	Yes	No
Distribution	VDss (human) (log L/kg)	−0.104	0.266	0.268	−0.288	0.375	0.201
	Fraction unbound (human) (Fu)	0.083	0	0	0.123	0.112	0.067
	BBB permeability (log BB)	−0.788	0.674	0.667	−0.911	−0.234	−1.192
	CNS permeability (log PS)	−3.135	−1.773	−1.773	−3.365	−1.816	−2.434
Metabolism	CYP2D6 substrate	No	No	No	No	No	No
	CYP3A4 substrate	Yes	Yes	Yes	Yes	No	No
	CYP1A2 inhibitor	Yes	No	No	Yes	Yes	Yes
	CYP2C19 inhibitor	Yes	No	No	No	Yes	No
	CYP2C9 inhibitor	Yes	No	No	No	Yes	Yes
	CYP2D6 inhibitor	No	No	No	No	Yes	No
	CYP3A4 inhibitor	No	No	No	No	Yes	Yes
Excretion	Total clearance (log mL/min/kg)	0.569	0.119	−0.044	2.04	0.076	0.458
	Renal OCT2 substrate	No	No	No	No	No	No
Toxicity	AMES toxicity	No	No	No	No	No	No
	Max. tolerated dose (human) (log mg/kg/day)	−0.048	−0.571	−0.56	0.193	0.286	0.82
	hERG I inhibitor	No	No	No	No	No	No
	hERG II inhibitor	No	Yes	Yes	No	Yes	No
	Oral rat acute toxicity (LD50) (mol/kg)	2.283	2.467	2.478	1.734	2.318	2.563
	Oral rat chronic toxicity (LOAEL) (log mg/kg_bw/day)	2.152	0.856	0.873	2.897	0.992	1.516
	Hepatotoxicity	No	No	No	No	No	No
	Skin sensitization	No	No	No	Yes	No	No
	*Tetrahymena pyriformis* toxicity (log ug/L)	0.371	0.384	0.383	0.617	0.434	0.323
	Minnow toxicity (log mM)	1.32	−1.309	−1.345	−0.969	−0.102	1.498
Drug-likeness	Bioavailability score (%)	0.55	0.55	0.55	0.55	0.55	0.55
	Lipinski’s Rule of Five	Yes; 0 violation	No; 2 violations: MW > 350, XLOGP3>3.5	No; 2 violations: MW > 350, XLOGP3>3.5	No; 3 violations: MW > 350, Rotors>7, XLOGP3>3.5	No; 1 violation: XLOGP3>3.5	Yes; 0 violation

## Discussion

This study revealed the first-time report on compound isolation ascertained by structure elucidation employing NMR technology from two *Colocasia* species, namely, *C. gigantea and C. affinis*. Previous reports on these plants only focused on phytochemical profiling through HPLC-DAD and GC-MS (Zilani et al., 2021; Alam et al., 2024). Thus, phytochemical isolation and the elucidation of their structures are the novelty of this current research. These revealed three bioactive flavonoids along with two triterpenoids and one monoglyceride in total, which sheds light on the polyphenol-rich candidacy of these plants, which may result in promising lead compounds for future drug discovery and development. Dichloromethane fractions of both plants were used in phytochemical screening and isolation of compounds. A total of four compounds were elucidated from the dichloromethane soluble fraction of *C. gigantea* as a result of consecutive chromatographic separation and purification. The compounds isolated from *C. gigantea* are penduletin (**1**), a mixture of α-amyrin (**2a**) and β-amyrin (**2b**), and a monoglyceride of stearic acid (**3**). Another three compounds were isolated from the dichloromethane-soluble fraction of *C. affinis* following the same technique: penduletin (**4**) (which was also isolated from *C. gigantea*), 7,8 -(3″,3″-dimethyl-pyrano)-4′-hydroxy flavonol (**5**), and a mixture of 7,8 -(3″,3″-dimethyl-pyrano)-4′-hydroxy flavonol (**5**) and 4′,7,8-trihydroxy flavonol (**6**).

The ^1^HNMR spectrum (500 MHz, CDCl3) of compounds **1** and **4** (Figure 4) in CDCl_3_ indicated the presence of three methoxy groups at δ_H_ 4.04 (3H, s), δ_H_ 4.02 (3H, s), and δ_H_ 3.90 (3H, s). The two pairs of *ortho*-coupled (*J =* 9.0 Hz) doublets at δ_H_ 7.04 and δ_H_ 7.90 showed that ring B is monosubstituted at C-4’. The peak at δ_H_ 12.79 belongs to 5-OH. There is a one-proton singlet at δ_H_ 6.59. There are three possible positions for this singlet: C-3, C-6, and C-8. Another proton singlet at δ_H_ 6.37 could be assigned to the -OH group at C-4’. These ^1^H NMR data are in close agreement with the published value of penduletin (Wang et al., 1989; Makhmoor and Choudhary, 2010), where the singlet at δ_H_ 6.59 is at the C-8 position, and the three methoxy groups are at the C-3, C-6, and C-7 positions. Thus, the structure of compounds **1** and **4** can be concluded as penduletin.

The ^1^H NMR spectrum (400 MHz, CDCl_3_) of compound **2** (Figure 5) showed two triplets at δ_H_ 5.32 ppm and δ_H_ 5.28 ppm characteristic of the olefinic proton (H-12) of α- and β-amyrin, respectively. In addition, eight singlets and two doublets of nCH_3_ protons were identified in the range δ_H_ 1.28-0.79 ppm. The singlet at δ_H_ 0.89 (3H) and δ_H_ 0.88 ppm (3H) indicated the presence of CH_3_-28. This means that the analyzed component was not an acid with the carboxyl group at C-17. The peaks of the methyl groups of α-amyrin were identified at δ_H_ 1.16 ppm (singlet; CH_3_-27), δ_H_ 0.93 ppm (doublet-doublet; *J =* 3.2 Hz; CH_3_-30), and δ_H_ 0.82 ppm (singlet; CH_3_-29). The protons of the CH_3_-27, CH_3_-29, and CH_3_-30 groups of β-amyrin had peaks at δ_H_ 1.28 ppm (singlet; 3H; CH_3_-27) and δ_H_ 0.95 ppm (singlet; 6H; CH_3_-29 and 30). The other signals were identical for both amyrins: δ_H_ 1.11 (6H, s; CH_3_-26), δ_H_ 1.01 (6H, s; CH_3_-28), δ_H_ 0.98 (6H, s; CH_3_-25), δ_H_ 0.82 (6H, s; CH_3_-23), and δ_H_ 0.80 (6H, s; CH_3_-24). The above data identified compound **2** as a mixture of α-amyrin (**2a**) and β-amyrin (**2b**) with a ratio of 4:3 based on the ^1^H NMR peak heights. This identity was further confirmed by a direct comparison of its ^1^H NMR spectrum with that recorded for a mixture of α-amyrin and β-amyrin in CDCl_3_ (400 MHz, CDCl_3_) (Migas et al., 2005).

The structure of compound **3** can be proposed as a monoglyceride of saturated fatty acid. The ^1^H NMR spectrum (400 MHz, CDCl_3_) showed signals at δ_H_ 4.195 (2H, dd, *J* = 13.6, 5.6 Hz), δ_H_ 3.957 (1H, m), and δ_H_ 3.732 (2H, dd, *J* = 11.6, 4.0 Hz). This group of signals can be attributed to the protons of the (-CH_2_-CH-CH_2_-) backbone (glycerol structure). The upfield peak at δ_H_ 0.903 with three proton intensities corresponds to one –CH_3_ proton. Two additional distinct groups of upfield signals at δ_H_ 2.374 (2H, t, *J* = 7.6 Hz), δ_H_ 1.64 (2H, m), δ_H_ 1.313 (8H, m), and δ_H_ 1.25 (20H, m) could correspond to 16 (sixteen) methylene groups. The presence of an additional oxymethine proton in the carbon chain can be justified by the signal at δ_H_ 3.617 (1H, dd, *J* = 11.6, 5.6 Hz). Comparing all the spectral data on the structure of compound **3** with the previously documented study (Kavadia et al., 2017) supports identifying this compound as a monoglyceride of stearic acid with the structure drawn in Figure 6.

The ^1^H NMR spectrum (CDCl3, 500 MHz) of compound **5** (Figure 7) showed two aromatic systems, including one 1,4-disubstituted benzene ring with the doublet at δ_H_ 7.68 (2H, d, *J =* 8.5 Hz), one tetra-substituted benzene ring with the ^1^H signals at δ_H_ 6.86 (1H, d, *J =* 8.5 Hz), δ_H_ 7.46 (1H, d, *J =* 8.5 Hz), and a 3″,3″-dimethyl-pyrano group. The presence of 3″, 3″-dimethyl-pyrano group can be proved by two tertiary methyl signals at δ_H_ 1.55 (6H, s) and two olefinic proton signals at δ_H_ 5.87 (1H, d, *J* = 12.5 Hz) and δ_H_ 6.96 (1H, d, *J =* 12.5 Hz). All these chemical shift values are in close agreement with the published values of citrusinol except for the presence of an extra aromatic proton signal that could be assigned to H-5. Another change in the chemical shift of the H-6 proton at δ_H_ 6.84 differs largely in values, which is at δ_H_ 6.21 for citrusinol (Qing-Hui et al., 2017). This high value of the chemical shift can be justified only if the position of the oxygen atom of the pyran ring is at C-8 rather than C-7. Thus, the structure of compound **5** can be proposed as 7, 8-(3″,3″-dimethyl-pyrano)-4′-hydroxy flavonol.

The ^1^H NMR spectrum (CDCl_3_, 500 MHz) of compound **6** shows signals that exactly match those of compound **5** (7,8-(3″,3″-dimethyl-pyrano)-4′-hydroxy flavonol). But the presence of additional peaks suggest the presence of another compound as a mixture. The additional signals include two para-coupled doublets with *J =* 8.5 Hz at δ_H_ 8.00 and δ_H_ 6.88, each integrating for two protons, which were assigned to the coupled H-2′ and H-6′, and H-3′ and H-5′, respectively. A typical ortho-coupled signal for protons is found at C-5 and C-6 at δ_H_ 7.46 (1H,d, *J =* 9.0 Hz) and δ_H_ 7.30 (1H,d, *J =* 9.0 Hz). All these additional proton signal values are in close agreement with published values (Ponce et al., 2009) of the structure (Figure 8) and suggested as 4′,7,8-trihydroxy flavonol. Thus, the structure of compound **6** appeared as a mixture of 4′,7,8-trihydroxy flavonol with 7,8-(3″,3″-dimethyl-pyrano)-4′-hydroxy flavonol (compound **5**) in a ratio of 1:2, according to the peak heights in the ^1^H NMR spectrum.

Diarrhea, characterized by disrupted intestinal movements and fluid accumulation coupled with increased peristalsis, occurs due to disruptions in the electrolyte permeability of the intestinal membrane (Bristy et al., 2020). Infectious diarrhea primarily arises from the invasion and spread of pathogens, particularly *Salmonella* and *Shigella* (Panda et al., 2012), against which traditional remedies have proven effective (Koné et al., 2004). The study of phytochemicals extracted from medicinal plants showcased promising antidiarrheal effects attributed to their various phytoconstituents, notably flavonoids, which are known for their antidiarrheal properties (Otshudi et al., 2000). Flavonoids are thought to achieve this by lessening motility in both the small and large intestine and suppressing bowel contractions (Capasso et al., 1991; Meli et al., 1990). All of the identified phytochemicals exhibited more pronounced antidiarrheal activity at higher doses (20 mg/kg b.w.) than at lower doses (10 mg/kg b.w.) by reducing the average weight of total and wet feces, as well as decreasing the total number of feces at a dose of 20 mg/kg, akin to the efficacy of loperamide, the standard drug used to treat diarrhea in this investigation, despite not displaying quite a dose-dependent response. However, unlike flavonoids and amyrin, the ester did not exert noteworthy antidiarrheal activity in mouse models.

The human body orchestrates a myriad of chemical reactions through various catabolic and anabolic processes, yielding a plethora of substances. These reactions, which can induce pain, inflammation, and oxidative stress, are often triggered by inflammatory mediators and reactive oxygen and nitrogen species (RONS) (Wilhelm et al., 2016). Inflammatory cascades, spurred by triggers such as microbial invasion or tissue distress, prompt the mobilization of defense cells like leukocytes to the affected site, a phenomenon orchestrated by receptors such as toll-like receptors (TLRs) and NOD-like receptors (NLRs) (Abdel Motaal and Abdel Maguid, 2005). Furthermore, an array of inflammatory mediators such as eicosanoids, cytokines, chemokines, and vasoactive amines, displays the inflammatory milieu (Sun et al., 2014). Flavonoids have demonstrated the capacity to curtail the abundance of inflammatory cells and the synthesis of MMP-9 (matrix metalloproteinase) and other inflammatory mediators (Li et al., 2012). Moreover, several flavonoids have demonstrated anti-inflammatory prowess by inhibiting the expression of the COX-2 gene (Chen et al., 2000). In the present study, the isolated flavonoids exhibited significant inhibition of writhing and licking compared to the standard. Hence, it can be conjectured that the secondary metabolites, notably flavonoids, may exert an analgesic effect by quelling the inflammatory cascade. Interestingly, other compounds, especially a mixture of α- and β- amyrin, also showed noteworthy activity. Previous studies also support the candidacy of the analgesic activity of amyrin through a prospective association of prostaglandins and TNF-alpha inhibition (Aragao et al., 2008). A monoester extracted from the plant showed relatively mild analgesic activity compared to other compounds, which invalidates its strong candidacy as a lead compound for further drug development exploration.

DHFR, an enzyme present in all living organisms, plays a vital role in cellular metabolic pathways. It facilitates the conversion of dihydrofolate to tetrahydrofolate using reduced nicotinamide adenine dinucleotide phosphate (NADPH) as a cofactor. Tetrahydrofolate is further utilized in the synthesis of thymidylate, purines, and amino acids through biosynthetic processes (Canh Pham and Truong, 2022). Thus, binding to DHFR and inhibition of this enzyme leads to the disruption of nucleic acid synthesis, ultimately causing cell death (Kalogris et al., 2014). Although ciprofloxacin primarily targets GyrA and ParC, docking ciprofloxacin with DHFR would be an exploratory computational exercise to see if there are any meaningful interactions between the drug and the enzyme (Dhillon et al., 2013). In the *in silico* study, ciprofloxacin showed a docking score of −8.1 kcal/mol with the DHFR enzyme, including carbon-hydrogen bond, pi-alkyl, and unfavorable donor-donor interactions with the respective amino acids (Table 6; Figure 12). Compounds **C5, C2a,** and **C2b** surpassed this value and gave docking scores of −9.1 kcal/mol, −8.8 kcal/mol, and −8.8 kcal/mol, respectively (Table 5). Among all the compounds, **C5** showed the highest binding affinity with interactions of pi-sigma, alkyl, pi-alkyl, and conventional hydrogen bonds (Table 6; Figure 9 **)**. The binding interactions for the other compounds are briefly depicted in Table 6. These binding interactions in molecular docking are important as they determine the strength and stability of the ligand binding to the target protein, which directly influences the efficacy of a potential drug or inhibitor (Morris and Lim-Wilby, 2008).

µ, κ, and δ are the opioid receptors that modulate the activity of the enteric nervous system and the release of neurotransmitters, thereby influencing both stimulatory and inhibitory motor pathways. This sequence of events influences gastrointestinal motility and stool consistency by slowing colonic transit, reducing the sensitivity of enteric nerves, and altering the secretion and transportation of fluids (Pannemans and Corsetti, 2018). Loperamide is an opioid agonist and serves as a potential antidiarrheal agent (Kopsky et al., 2019). In computational docking studies, for antidiarrheal activity, the standard loperamide drug molecule showed a binding affinity of −9.1 kcal/mol with the KOR receptor, including pi-sigma, pi-pi stacked, and alkyl bond interactions (Tables 5, 6; Figure 12). Compounds **C2b**, **C5,** and **C2a** had higher docking scores of −10.5 kcal/mol, −9.7 kcal/mol, and −9.4 kcal/mol, respectively, compared to the standard drug (Table 5). The compound β-amyrin **(C2b)** exhibited the highest affinity among all the compounds, including alkyl bond interactions with the amino acids Tyr 140, Ile 180, Trp 183, Leu 184, and Ile 191 (Table 5; Figure 10). Considering these binding interactions is for in predicting the binding affinity and stability of a ligand–protein complex, which ultimately affects the design of more effective drugs. Effective docking must consider these interactions to identify strong binders with good pharmacological potential (Morris and Lim-Wilby, 2008).

The identified compounds were docked against the COX-2 enzyme to investigate the peripheral analgesic properties. The prostaglandins synthesized from arachidonic acids by the COX-2 enzyme are responsible for the sensation of pain and inflammation (Lee et al., 2005). Therefore, COX-2 inhibitors are considered to work in chronic pain relief and post-surgical analgesia by reducing the production of prostaglandins responsible for inflammation, pain, and fever, along with the minimization of gastrointestinal side effects (Camu et al., 2003). Diclofenac sodium is a well-established COX-2 inhibitor, and it interferes with the production of prostaglandin G2, which is the precursor to other prostaglandins (Kaur and Sanyal, 2011). In computational docking modeling, this standard drug showed a binding affinity of −7.8 kcal/mol against the COX-2 enzyme, including pi-sigma, amide-pi stacked, pi-alkyl, and conventional hydrogen bond interactions (Tables 5, 6; Figure 12). The compound β-amyrin **(C2b)** showed a higher docking score of −8.7 kcal/mol than the standard and demonstrated a strong conventional hydrogen bond interaction with the amino acid Asp 347 (Tables 5, 6; Figure 11). Compounds **C5** and **C6** also exhibited notable binding affinities of −7.2 kcal/mol and −7.3 kcal/mol, respectively. These interactions are crucial for specific binding, as they enhance the affinity and selectivity of the ligand toward the target. The interactions improve docking scores, lead to more stable complexes, and play a key role in fine-tuning the fit of the ligand within the binding pocket, ensuring proper spatial alignment (Morris and Lim-Wilby, 2008).

The ADME/T study of the identified compounds also exhibited promising results, which are listed in Table 7. According to Lipinski, if the following requirements, such as molecular weight <500 amu, hydrogen bond acceptor sites <10, hydrogen bond donor sites <5, and lipophilicity value LogP ≤5 are met, then that particular substance is likely to be orally active (Zhang and Wilkinson, 2007). Compounds penduletin **(C1/C4)** and 4′,7,8-trihydroxy flavonol **(C6)** did not violate the five Lipinski principles. Additionally, 7,8-(3″,3″-dimethyl-pyrano)-4′-hydroxy flavonol **(C5)** violated only one of Lipinski’s rules. However, the compounds α- and β-amyrin **(C2a and C2b)** each had two violations, and the monoglyceride of stearic acid **(C3)** compound had three violations. The results suggest that the compounds **C1/C4** and **C5** conform to Lipinski standards, signifying their oral safety and potential as promising candidates for medicinal purposes.

pkCSM is a novel approach to predicting pharmacokinetic and toxicological consequences that use graph-based signatures to represent the chemistry and topology of small compounds (Pires et al., 2015). Table 7 shows that all the test compounds possessed negative water solubility values (log mol/L), suggesting that they are lipophilic, which promotes effective absorption (Kuentz and Arnold, 2009). The bioavailability scores of all compounds were found to be 0.55%. None of the identified test compounds exhibited any hepatotoxicity. Moreover, none of the compounds was found to inhibit hERG I, and except for **C2a**, **C2b,** and **C5**, none of them inhibited hERG II, indicating that these compounds may not be cardiotoxic (Muster et al., 2008).

Finally, from all the hypotheses of the *in silico* study, the identified compounds that gave promising docking scores with the respective receptors, adhered to Lipinski’s rule, and conformed satisfactorily with pharmacokinetic and toxicological assumptions should be considered for their drug candidacy and further investigation. Compared with existing therapeutic agents, the identified compounds exhibited remarkable effects in bioassays as well as remarkable binding affinities with the corresponding biomolecules.

## Conclusion

Vegetables are a source of nutrition and disease management and can be crucial as nutritional strategies for integrative healthcare. The discovery of promising pharmacological activities and bioactive phytochemicals in locally consumed vegetables can add a new dimension to therapeutics, pharmacological research, and development. The present study represents the first-time report on compound isolation from *C. gigantea* and *C. affinis*, employing NMR technique. The outcome reveals three bioactive flavonoids, two triterpenoids, and a monoglyceride, underscoring these species’ polyphenol-rich attributes. Upon biological investigations, these compounds showed prospective antimicrobial, analgesic, and antidiarrheal potentials, justifying the tribal use of these taro vegetables. Further extensive scientific research on the isolated compounds in dosage form on a clinical trial basis is highly recommended to ascertain their safety and efficacy profile and mode of action to ease the process of future drug discovery and development.

## Data Availability

The original contributions presented in the study are included in the article/supplementary material; further inquiries can be directed to the corresponding author.
