# Corrigendum: First-time report on compound isolation from two *Colocasia* species: vegetable-derived bioactive metabolites and their medicinal potential

**DOI:** 10.3389/fphar.2025.1541225

**Published:** 2025-04-11

**Authors:** Safaet Alam, Fahmida Tasnim Richi, Nazim Uddin Emon, Abu Asad Chowdhury, Choudhury Mahmood Hasan, Mohammad Rashedul Haque

**Affiliations:** ^1^ Chemical Research Division, BCSIR Dhaka Laboratories, Bangladesh Council of Scientific and Industrial Research (BCSIR), Dhaka, Bangladesh; ^2^ Department of Pharmaceutical Chemistry, Faculty of Pharmacy, University of Dhaka, Dhaka, Bangladesh; ^3^ Department of Pharmacy, Faculty of Science and Engineering, International Islamic University Chittagong, Chittagong, Bangladesh

**Keywords:** *Colocasia gigantea*, *Colocasia affinis*, vegetable, NMR, antibacterial, antidiarrheal, analgesic, anti-inflammatory

In the published article, there was an error in the **Footnote** for “Table 3” as published. An incorrect expression was used; “[n = 5]” was used when this should have been “[n = 3]”. The corrected footnote appears below.

“Values are expressed as mean ± SEM (n = 3); CTL, negative control; STD, positive control; ***p < 0.001, **p < 0.01, *p < 0.05 compared to negative control.”

In the published article, there was an error in Table 4 as published. Columns 1-3 of “Table 3” were incorrectly replicated as Table 4. The corrected Table 4 and its caption appear below.

**TABLE 4:** Anti-inflammatory effect of the isolated compounds from *C. gigantea* and *C. affinis* on the formalin-induced mouse model.

Animal group with respective doses (ml/kg or mg/kg, b.w; p.o)	Time of licking in seconds (Ealy Phase; 0–5 min)	Time of licking in seconds (Late Phase; 15–30 min)
CTL	60.38 ± 2.20	73.68 ± 1.34
STD (Ibuprofen, 10 mg/kg, b.w)	15.98 ± 1.62^***^	18.65 ± 2.52^***^
Compound 1 or 4 (10 mg/kg, b.w.)	32.54 ± 3.12^***^	38.87 ± 1.09^***^
Compound 1 or 4 (20 mg/kg, b.w.)	31.38 ± 1.12^***^	27.61 ± 1.02^***^
Compound 2 (10 mg/kg, b.w.)	44.32 ± 1.52^***^	51.49 ± 0.71^***^
Compound 2 (20 mg/kg, b.w.)	19.23 ± 3.34^***^	35.32 ± 1.77^***^
Compound 3 (10 mg/kg, b.w.)	38.04 ± 4.19^**^	45.68 ± 1.95^***^
Compound 3 (20 mg/kg, b.w.)	28.43 ± 2.38^***^	30.56 ± 2.16^***^
Compound 5 (10 mg/kg, b.w.)	40.38 ± 1.51^***^	53.29 ± 1.43^***^
Compound 5 (20 mg/kg, b.w.)	21.43 ± 0.53^***^	24.56 ± 0.98^***^
Mixture of compound 5 and 6 (10 mg/kg, b.w.)	44.28 ± 2.20^**^	56.21 ± 2.40^**^
Mixture of compound 5 and 6 (20 mg/kg, b.w.)	30.44 ± 1.05^***^	36.43 ± 4.81^**^

In the published article, there was an error. The number of mice included in the control and test groups for pharmacological assessment was incorrect.

A correction has been made to **Materials and Methods**, *Experimental design*, *In vivo* tests, *Antidiarrheal bioassay: castor oil-induced diarrhea test*, Paragraph 1. This sentence previously stated:

“In this study, the mice were divided into four groups: control, positive control, and two test groups, each containing five mice.”

The corrected sentence appears below:

“In this study, the mice were divided into twelve groups: control, positive control, and ten test groups, each containing three mice.”

In the published article, there was an error. The number of mice included in the control and test groups for pharmacological assessment was incorrect.

A correction has been made to **Materials and Methods**, *Experimental design*, *In vivo* tests, *Analgesic bioassay: acetic acid-induced writhing test*, Paragraph 1. This sentence previously stated:

“In this study protocol, mice were divided into four groups: control, positive control, and two test groups, each containing five mice.”

The corrected sentence appears below:

“In this study protocol, mice were divided into twelve groups: control, positive control, and ten test groups, each containing three mice.”

In the published article, there was an error.

A correction has been made to **Results**, *In vivo* study, *Effect of the identified test compounds on formalin-induced licking in the mice model*, Paragraph 1. These sentences previously stated:

“C5 and C2 demonstrated the highest anti-inflammatory efficacy among the compounds evaluated, resulting in a 44.44% and 40.74% reduction in licking respectively, at a dose of 20 mg/kg. On the other hand, the standard drug ibuprofen led to a decrease of 77.78% at a dose of 10 mg/kg.”

The corrected sentences appear below:

“C5 and C2 demonstrated the highest anti-inflammatory efficacy among the compounds evaluated, resulting in a 64.50% and 68.15% reduction in licking during the early phase and a 66.66% and 52.06% reduction during the later phase, respectively, at a dosage of 20 mg/kg. On the other hand, the standard drug ibuprofen led to a decrease of 73.54% and 74.68% in early and late phase subsequently.”

The authors apologize for these errors and state that this does not change the scientific conclusions of the article in any way. The original article has been updated.

